# Fraction of Exhaled Nitric Oxide (**Fe_NO_**) Norms in Healthy Tunisian Adults

**DOI:** 10.1155/2014/269670

**Published:** 2014-06-03

**Authors:** Sonia Rouatbi, Mohamed Ali Chouchene, Ines Sfaxi, Mohamed Ben Rejeb, Zouhair Tabka, Helmi Ben Saad

**Affiliations:** ^1^Department of Physiology and Functional Explorations, Farhat HACHED Hospital, 4000 Sousse, Tunisia; ^2^Laboratory of Physiology, Faculty of Medicine of Sousse, University of Sousse, 4000 Sousse, Tunisia; ^3^Department of Prevention and Care Safety, Sahloul Hospital, 4000 Sousse, Tunisia; ^4^Research Unit: Secondary Prevention after Myocardial Infarction, N: 04/UR/08-18, Faculty of Medicine of Sousse, University of Sousse, 4000 Sousse, Tunisia

## Abstract

*Aims*. To establish Fe_NO_ norms for healthy Tunisian adults aged 18–60 years and to prospectively assess their reliability. * Methods*. This was a cross-sectional analytical study. A convenience sample of healthy Tunisian adults was recruited. Subjects responded to a medical questionnaire, and then Fe_NO_ levels were measured by an online method (Medisoft, Sorinnes (Dinant), Belgium). Clinical, anthropometric, and plethysmographic data were collected. All analyses were performed on natural logarithm values of Fe_NO_. * Results*. 257 adults (145 males) were retained. The proposed reference equation to predict Fe_NO_ value is lnFe_NO_ (ppb) = 3.47−0.56× height (m). After the predicted Fe_NO_ value for a given adult was computed, the upper limit of normal could be obtained by adding 0.60 ppb. The mean ± SD (minimum-maximum) of Fe_NO_ (ppb) for the total sample was 13.54 ± 4.87 (5.00–26.00). For Tunisian and Arab adults of any age and height, any Fe_NO_ value greater than 26.00 ppb may be considered abnormal. Finally, in an additional group of adults prospectively assessed, we found no adult with a Fe_NO_ higher than 26.00 ppb. * Conclusion*. The present Fe_NO_ norms enrich the global repository of Fe_NO_ norms that the clinician can use to choose the most appropriate norms.

## 1. Introduction

The measurement of the fraction of nitric oxide in exhaled breath (Fe_NO_) is recognized as an accurate, reproducible, and completely noninvasive diagnostic test for airway disease [1]. In 2011, the American Thoracic Society (ATS) recommended that measuring Fe_NO_ can help diagnose eosinophilic airway inflammation, determine the likelihood of corticosteroid responsiveness and the potential need for corticosteroids, unmask unsuspected nonadherence to corticosteroid therapy, and aid asthma assessment [2].

In health, the Fe_NO_ largely derives from the lower respiratory tract, particularly the airways of the lung, if nasal air is excluded [3]. NO can be detected in exhaled air by several methods such as chemiluminescence, spectroscopy, electrochemical portable, and other methods currently under development [4]. Cheaper and easy to use [1], Fe_NO_ analyzers are now readily available and increasingly used not only for the diagnosis of eosinophilic airway inflammation which is seen mainly in asthma [5] but also for its assessment [6]. In addition, the ATS/European Respiratory Society (ATS/ERS) has jointly demonstrated that some factors (i.e., age, sex, and race) may affect the Fe_NO_ values [1].

Interpretation of Fe_NO_ data relies upon comparison of measured values with predicted ones available from published norms (e.g., fixed values, reference equations or normal values tables) [1, 7, 8]. However, to the best of our knowledge, Fe_NO_ norms are available only for some adult populations, mainly for Caucasians ones [9–24]. These norms can be used in clinical practice, provided that the study characteristics (population, sampling, and objective measures) are taken into consideration when such an equation is used for the interpretation of Fe_NO_ values [1, 7]. The published norms [9–24] differ considerably in terms of individual-specific factors that have an effect on Fe_NO_ values, and there is little standardization of the method description in the studies, both on the statistical and technical sides [7]. In addition, neither of these studies provided prospective verification for their studied populations nor proposed a clear method of interpreting the measured Fe_NO_ (e.g., using an upper-limit-of-normal (ULN) or a fixed percentage above which Fe_NO_ values would be considered abnormal). In addition, only few studies have included a comparison group [12, 13, 18, 21].

Recently, the Fe_NO_ of a large group of healthy Tunisian/Arab children was prospectively measured [25]. It was shown that the available published children Fe_NO_ norms did not reliably predict Fe_NO_ in this population [25]. Thus, a table of normal values according to age ranges was established. In addition, the need of reference equations specific to Tunisian/Arab adults' populations has been demonstrated for several lung function parameters [26–33], but not for Fe_NO_. Furthermore, and to the best of our knowledge, Fe_NO_ norms are established only in one Arabic population (Saudi Arabian males' [16]) and the applicability and reliability of these norms [16] should be assessed as regards to Tunisian Arab adults, in order to avoid erroneous clinical interpretation of Fe_NO_ data in this population. Moreover, the ATS/ERS has encouraged investigators to publish physiological norms for healthy populations of various racial backgrounds to enable individual subject results to be compared with data from a racially similar population [1]. The use of the same kind of assessment equipment and procedure is also recommended [1]. Therefore, the present study aims

1. to identify factors that influence the Fe_NO_ values of healthy Tunisian adults aged 18–60 years,
2. to test the applicability and reliability of the previously published Fe_NO_ norms for Saudi Arabian males [16] (the null hypothesis is that there will be no difference between measured and predicted Fe_NO_ mean values),
3. if needed to establish Fe_NO_ norms and to prospectively assess their reliability.

## 2. Methods

### 2.1. Study Design

The present study is a cross-sectional one spread over 7 months (May–December 2012). It was conducted at the Department of Physiology and Functional Explorations (Farhat HACHED Hospital, Sousse, Tunisia).

Study design consists of a convenience sample of healthy Tunisian adults aged 18–60 years (Arab race) in the region of Sousse.

Study approval was obtained from the hospital ethics committee and written informed consent was obtained from all subjects.

Detailed information about the study design appears in the Supplemental Data available online at http://dx.doi.org/10.1155/2014/269670.

### 2.2. Sample Size

It was calculated according to the following predictive equation [34]: *n* = (*Z* ^2^ *Pq*)/Δ^2^, where “*n*” was the number of required adult, “*Z*” was the 95% confidence level (=1.96), “*q*” was equal to “1 − *P*”, “Δ” was the precision (= 6%), and “*P*” was the estimation of adults aged more than 18 years with a normal Fe_NO_ value. Among the 298 participants (aged 25–75 years) who performed Fe_NO_ measurements, only 193 adults (*P* = 0.65) were categorized as normal by Travers et al. [13]. Plugging this relevant value into the predictive equation, the sample size was thus 243 adults. Therefore, to establish Fe_NO_ norms, we recruited an initial group (*equation group*) of 257 adults (145 females).

To verify the reliability of the present study norms, Fe_NO_ data were prospectively measured in a second group (*validation group*) of 50 additional healthy adults (25 females) meeting the inclusion criteria of the present study but not having participated in the first part.

### 2.3. Subjects

Volunteer healthy adults were included.

The following noninclusion criteria were applied: hay fever or chronic illnesses especially cardiovascular, renal, gastrointestinal, or neurological diseases; otorhinolaryngologic diseases or symptoms (allergic rhinitis, recurrent symptoms or rhinitis, symptoms and signs of acute upper respiratory infection during two weeks prior to assessment, and recent airway infection (cold, flu, and sore throat within the last seven days)); clinical manifestation of allergic diseases (urticaria, skin allergy, atopic dermatitis, or eczema); a history of pulmonary diseases or related respiratory symptoms (history of asthma or asthma medication use, current or past symptoms of wheeze or chronic cough, and chronic obstructive pulmonary disease); abnormal lung function data; pregnant at the time of assessment; regular medication (glucocorticoid, bronchodilator, leukotriene receptor agonist, antihistamine, etc.) use except contraceptive; current or ex-smokers (cigarettes or narghile use [35, 36]) and inability to perform properly Fe_NO_ or plethysmography measurements.

### 2.4. Medical Questionnaire and Physical Examination

A medical questionnaire [37] was used to assess several subject characteristics.

Age (yrs) was taken as the number of complete years from birth to the date of the study. Height (±0.01 m) and weight (±1 kg) were measured with a height gauge with shoes removed, heels joined, and back straight and subject without heavy clothes. Body mass index (BMI) was calculated (= weight/height^2^). Two groups of subjects were defined [38] nonobese (BMI < 30); obese (BMI ≥ 30). Body surface area (BSA, m^2^) was calculated [39].

#### 2.4.1. Fe_NO_ Measurement

The Fe_NO_ (parts per billion, ppb) was measured by Medisoft HypAir Fe_NO_ method using an electrochemical analyzer (Medisoft, Sorinnes (Dinant), Belgium). The instrument was calibrated and used according to the manufacturer's instructions and work in conjunction with a personal computer. The software supplied by either manufacturer provided visual feedback allowing the participant to maintain a constant exhaled breath flow rate. Measurements were made between 8 a.m. and 12 a.m.

The online method with constant flow rate was used [1]. After a full unforced exhalation outside the mouthpiece, a maximal inspiration was performed through an absorber to ensure NO-free air. The adult then performed a controlled exhalation using flow control at an exhalation pressure of 4–10 cm H_2_O for at least six seconds, during which time sample collection and gas analysis were performed. Nasal contamination is presented by closure of the velum by using five cm H_2_O oral back pressures. A nose clip was not used.

Subjects were asked not to eat, not to drink water or alcohol, and not to ingest caffeine nor participate in strenuous activities for two hours prior to the test [1].

Three acceptable measurements (within 10%) were taken at the recommended flow rate of 50 mL/s within a 15-minute period [1].

#### 2.4.2. Plethysmography Measurements

They were performed according to international guidelines [40] using a plethysmograph (ZAN 500, Me*β*greräte GmbH, Germany). Tests were made after the Fe_NO_ measurement [41].

The following parameters were measured/calculated: peak expiratory flow (PEF); forced vital capacity (FVC, L); 1st second forced expiratory volume (FEV_1_, L); maximal mid expiratory flow (MMEF, L/s) or forced expiratory flow when *x*% of FVC has been exhaled (MEF*x*, L/s); FEV_1_/FVC ratio (absolute value); total lung capacity (TLC, L); residual volume (RV, L); and thoracic gas volume (TGV, L). The results were compared with local age- and sex-matched reference values [42].

Obstructive or restrictive ventilatory defects were retained when, respectively, the FEV_1_/FVC ratio or the TLC was lower than the lower limit of normal (LLN) [40]. FEV_1_ and FVC were considered as abnormal when they were lower than the LLN [40].

### 2.5. Statistical Analysis

For each subject, the mean of the three correct Fe_NO_ values was used for statistical analysis.

Preliminary descriptive analysis included frequencies for categorical variables (sex: male/female) and obesity status (nonobese/obese) and means ± standard deviation (SD) and 95% confidence interval (95% CI) for continuous ones (anthropometric and plethysmographic data).

Since the distribution of the dependent variable (Fe_NO_) was log-normally distributed (Shapiro-Wilk test [43]), all analyses were performed on natural logarithm values of Fe_NO_ (lnFe_NO_). Fe_NO_ results were presented as geometric mean ± SD (95% CI, LLN to ULN) and as minimum-maximum.

*Comparison with Published F* *e*_*NO*_ *Norms for Saudi Arabian Males [16].* Habib et al. [16] developed two linear models (Box 1) for 121 Saudi Arabian males aged 19–64 years. More details about these norms are exposed in Supplemental Table 1. Individually measured present study males' Fe_NO_ was compared with the predicted Fe_NO_ from the two reference equations [16] for the same age range, using paired *t*-tests and scatter plots. Limits of agreement (measured-predicted) were calculated. If the Saudi Arabian reference equations provide limits of agreement closest to zero, they will be appropriate for the present population [40].

It is well known that Fe_NO_ values obtained with different devices are not directly comparable [44]. As the Aerocrine devices are much more commonly used and most of the other devices give pretty similar results [44] and as measurements on the HypAir Fe_NO_ are 1.6 times higher than those obtained with the Aerocrine NIOX [45] and for a better interpretation of the present study data, results were adjusted in accordance with Brooks et al. [45]. For that reason Fe_NO_ predicted values from Habib et al. [16] norms were divided by 1.6 and individually measured Fe_NO_ were compared with the predicted/adjusted Fe_NO_ from Habib et al. [16] norms as described above.

Student's *t*-tests were used to evaluate the associations between Fe_NO_ and the categorical variables. Pearson product-moment correlation coefficients evaluated the associations between Fe_NO_ and the continuous measures. The linearity of association between Fe_NO_ and the continuous measures was checked graphically by plotting each regressor against the Fe_NO_. Only significantly and linearly associated variables were entered into the model. A linear regression model was used to evaluate the independent variables explaining the variance in Fe_NO_. Candidate variables were stepped into the model with a stepwise selection method. To determine entry and removal from the model, significance levels of 0.15 and 0.05 were used, respectively. No colinearity between predictors was detected with variance inflation factors. The linearity was evaluated by correlation (*r*) and determination (*r* ^2^) coefficients and the standard error. The 95% CI (= 1.64 × residual-SD (RSD)) was calculated [43]. Detailed information about the natural logarithm linear regression equation appears in the Supplemental Data. 

*Fe*_*NO*_ *Reference Equations and Normal Values*. Three Fe_NO_ reference equations (for males, females, and total sample) were established, using only previously correlated factors in a stepwise linear regression model. A measured Fe_NO_ higher than the ULN (ULN = reference value + 1.64 × RSD) will be considered as abnormal.

A table for each age and height ranges for the total sample, presenting Fe_NO_ geometric mean ± SD (95% CI, LLN to ULN and minimum-maximum) is provided. Three ways are proposed to interpret a measured Fe_NO_ value.

- Use of the total sample Fe_NO_ maximum value as a threshold: each adult Fe_NO_ value higher than the total sample Fe_NO_ maximum value will be considered as abnormal.
- Use of a specific threshold (Fe_NO_ maximum value) for each age or height ranges: each Fe_NO_ value higher than these ages or height ranges Fe_NO_ maximum values will be considered as abnormal.
- Use of a specific threshold (Fe_NO_ maximum value) for each age and height range: each Fe_NO_ value higher than this age and height range Fe_NO_ maximum value will be considered as abnormal.

*Reliability of the Arab Tunisian* *Fe*_*NO*_ *Norms.* It was evaluated in the validation group in two ways. Fe_NO_ predicted normal values will be considered as reliable when no subject from the validation group will have a measured abnormal Fe_NO_ value (higher than predicted Fe_NO_ maximum value for each age and height ranges). The correlation between the measured Fe_NO_ values and those predicted by the Fe_NO_ reference equations is evaluated. The number of subjects having a measured Fe_NO_ value higher than the ULN is determined.

Analyses were carried out using Statistica (Statistica Kernel version 6, StatSoft, 26 France). Significance was set at the 0.05 level.

## 3. Results

### 3.1. Subject's Data

An initial sample of 400 voluntary adults of Arab race was examined. Noninclusion criteria, presented in detail in the Supplemental Data, were found in 93 subjects.

Two hundred and fifty-seven adults (equation group) were included to establish Fe_NO_ norms and 50 adults were included as a validation group.

Between the males and females of the equation group (Table 1 and Supplemental Table 3), there was a significant difference in anthropometric data (age, weight, height, and BSA) and plethysmographic data expressed in absolute values (exposed in Supplemental Table 3) (FVC, FEV_1_, FEV_1_/FVC, PEF, MMEF, MEF_50_, MEF_75_, TLC, TGV, and RV) or expressed as a percentage of predicted values (FVC, FEV_1_, MMEF, TLC, and TGV). In addition, significantly higher females were categorized as obese. No statistical significant difference was found between females' and males' means Fe_NO_ data, respectively, 13.31 ± 4.55 versus 13.84 ± 5.26 ppb.

Supplemental Figure 1 shows the distribution of the 257 healthy adults according to sex and age range. Compared to females, there was a significantly lower number of males aged 17–35 years and a significantly higher number of males aged 45–55 years.

Supplemental Figure 2 shows the distribution of the adults Fe_NO_ data according to age, height, and weight ranges. A significant Fe_NO_ difference was found between subjects at the height of 1.36–1.55 m.

### 3.2. Univariate Analysis

Sex (Table 1) and obesity status (Table 2) did not significantly affect the Fe_NO_ value.

For the total sample, Fe_NO_ was significantly correlated with height and TGV (%). For males, Fe_NO_ was significantly correlated only with height. For females, Fe_NO_ was significantly correlated with height and some plethysmographic data (FVC (L), FEV_1_ (L), PEF (L/s), MMEF (L/s, %), MEF_25_ (L/s), MEF_50_ (L/s, %), MEF_75_ (L/s, %), and TGV (L)) (Table 2).

### 3.3. Multivariate Analysis (Fe_NO_ Influencing Factors, Table 3)

For females, height (m), MEF_50_ (%), and TGV (L) explained a slight (*r* ^2^ = 9.24%) but significant Fe_NO_ variability. For males and the total sample, only height (m) explained a slight (resp., *r* ^2^ = 3.80% and *r* ^2^ = 1.92%) but significant Fe_NO_ variability. The retained Fe_NO_ reference equation is exposed in Box 2.

### 3.4. Comparison, without Values Adjustment according to Brooks et al. [45], with Published Fe_NO_ Norms for Saudi Arabian Males [16]

**Figure 1:**
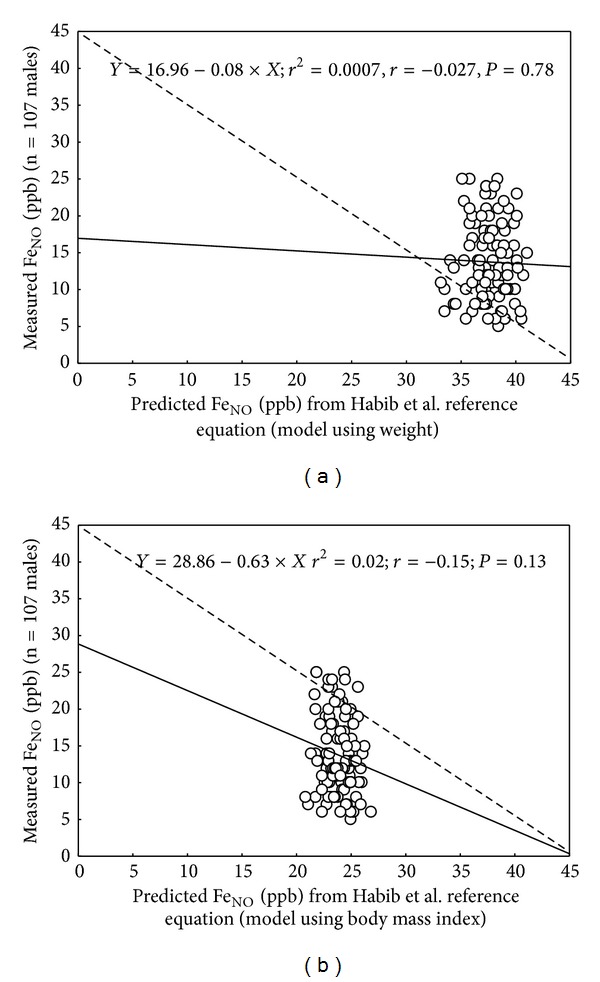
Comparison, for the same age range, of measured and predicted fraction-of-exhaled-nitric-oxide (Fe_NO_) determined from Saudi Arabian norms: (a) model including weight. (b) Model including body mass index. *n* = number of males having the age range of the Saudi Arabian predicted Fe_NO_ study. Solid line (—): regression line. Dashed line (- - -): identity line. *r* ^2^: coefficient of determination. *r*: correlation coefficient. *P*: probability.

Figure 1 shows individually measured Fe_NO_ plotted against the corresponding predicted value for the same age range, using the Saudi Arabian model 1 (Figure 1(a)) or model 2 (Figure 1(b)) reference equations. As can be seen, the data showed wide disparity compared to the identity line with a systematic bias between the measured and predicted values. In addition, the present study mean ± SD measured Fe_NO_ was significantly overestimated by 23.95 ± 5.58 ppb and by 10.12 ± 5.60 ppb, with, respectively, the model 1 (Figure 1(a)) and the model 2 (Figure 1(b)) reference equations.

### 3.5. Comparison, after Values Adjustment according to Brooks et al. [45], with Published Fe_NO_ Norms for Saudi Arabian Males [16]

Supplemental Figure 3 shows individually measured Fe_NO_ plotted against the corresponding predicted/adjusted value for the same age range, using the Saudi Arabian model 1 (Supplemental Figure 3(a)) or model 2 (Supplemental Figure 3(b)) reference equations. The data still showed disparity compared to the identity line with a systematic bias between the measured and predicted/adjusted values. The present study mean ± SD measured Fe_NO_ was significantly overestimated by 9.82 ± 5.41 ppb (*P* < 0.05) and only by 1.18 ± 5.45 ppb (*P* = 0.02), with, respectively, the model 1 (Supplemental Figure 3(a)) and the model 2 (Supplemental Figure 3(b)) reference equations.

### 3.6. Tunisian Adults Fe_NO_ Norms (Fe_NO_ Reference Equation or Table Norms)

Due to the inadequacy of the Saudi Arabian males' Fe_NO_ reference equations [16], norms adapted to Tunisian population were established.

For a practical interest, and as sex did not significantly affect the Fe_NO_ value, authors recommend the use of the total sample reference equation (Box 2), when calculating a predicted Fe_NO_ value. The latter explains almost 2% of the Fe_NO_ variability. After the predicted Fe_NO_ value for a given adult was computed from this equation, the ULN could be obtained by adding 0.5992724 ppb.

Since the correlation between height and Fe_NO_ was very slight, Fe_NO_ normal values for Arab Tunisian adults aged 18–60 years were developed, taking into consideration age and height ranges. These Fe_NO_ normal values are presented as geometric mean ± SD and minimum-maximum (Table 4). It is much simpler for clinicians to remember and device manufacturers to program. In practice, three ways can be used to interpret a measured Fe_NO_ value.

- Use of the total sample Fe_NO_ maximum value as a threshold: each adult Fe_NO_ value higher than 26 ppb will be considered as abnormal.
- Use of a specific threshold (Fe_NO_ maximum value) for each age or height range: for example, for a given adult aged 17–35 years each Fe_NO_ value higher than 24 ppb will be considered as abnormal and for a given adult having a height range from 1.36 to 1.55 m, each Fe_NO_ value higher than 24 ppb will be considered as abnormal.
- Use of a specific threshold (Fe_NO_ maximum value) for each age and height range: for example, for a given adult aged 17–35 years having a height range from 1.36 to 1.55 m, each Fe_NO_ value higher than 22 ppb will be considered as abnormal.

### 3.7. Reliability of Tunisian Fe_NO_ Norms

The mean ± SD age, height, weight, and BMI of the validity group were, respectively, 40.88 ± 13.45 years, 1.66 ± 0.09 m, 75 ± 22 kg, and 28 ± 9 kg/m^2^. The validation group anthropometric data are similar to those of the equation (Supplemental Table 4). However, significant differences were noted for FVC, FEV_1_ and RV expressed as percentages of predicted values (Supplemental Table 4). Supplemental Figure 4 exposes the measured Fe_NO_ values of the equation and validation groups according to height. The validation group Fe_NO_ values are closer to those of the equation.

**Figure 2:**
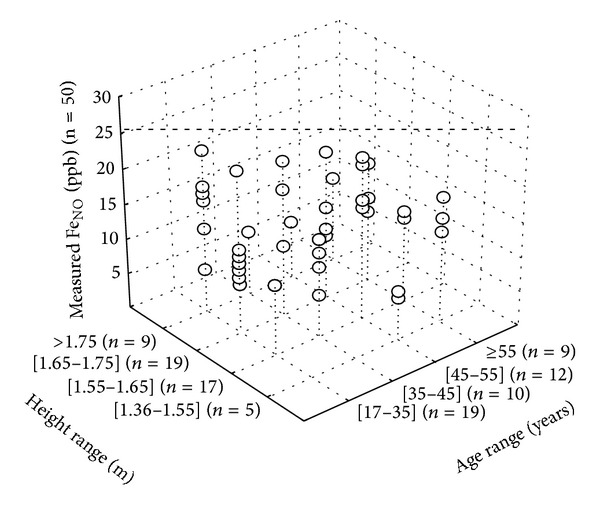
Three-dimension presentation (*XYZ* scatterplot) of the validity group measured fraction-of-exhaled-nitric-oxide (Fe_NO_) values. Fe_NO_ (*Y*-axis) versus age (*X*-axis) and height (*Z*-axis) ranges described in Table 4. *n* = number of healthy Arab Tunisian subjects. Dashed line (- - -): predicted Fe_NO_ maximum value for the total sample (=26 ppb).

The validation group measured Fe_NO_ values are shown in Figure 2. The application of the normal values mentioned in Table 4 found no adult with a measured Fe_NO_ higher than the predicted specific threshold for each age and height range. In addition, no adult had a measured Fe_NO_ value higher than the predicted total sample Fe_NO_ maximum value (=26 ppb).

The geometric mean ± SD (minimum-maximum) Fe_NO_ prospectively measured was 12 ± 5 (6–23) ppb. When expressed as a percentage of predicted value derived from the total sample reference equation (Box 2), the geometric mean ± SD (minimum-maximum) of the Fe_NO_ was 96 ± 39% (44–203).

## 4. Discussion

The Fe_NO_ of a large group of healthy Tunisian/Arab adults aged 18–60 years old was prospectively measured. The Fe_NO_ norms for Saudi Arabian did not reliably predict Fe_NO_ in the local population and Fe_NO_ values are lower in healthy Tunisian/Arab adults than in Saudi Arabian population. So, the null hypothesis that we would see no difference in the means of the measured and predicted Fe_NO_ mean values was rejected. Thus, a table of normal values according to age and height ranges was established. For Arab Tunisian adults of any age and height, any Fe_NO_ value greater than 26 ppb may be considered abnormal. In addition a reference equation taking into consideration height was established. Finally, in an additional group of 50 adults prospectively assessed, no adult with a Fe_NO_ higher than the threshold of 26 ppb or higher than the 95% CI ULN specific for each age and height ranges was found.

### 4.1. Subject's Data

As for almost all the studies aiming to publish Fe_NO_ norms [9–23] the present study was a convenience sample.

The recruitment mode and adult age range were similar to previous studies having comparable aims to the present one [9, 10, 12, 14, 16, 20, 22].

The present study which calculated sample size (*n* = 257) “seemed” to be satisfactory. The retained Fe_NO_ reference equation allowed the explanation of 2% of the Fe_NO_ variability, which appears to be less than reported data (*r* ^2^ ranged 6% [22] to 34% [10]).

Fe_NO_ was prospectively measured in a validation group of additional healthy adults meeting the inclusion criteria of the present study. To the best of our knowledge, the present study is the first one that uses a validation healthy group to verify the reliability of the retained Fe_NO_ norms.

Similar to some studies [9, 10, 15, 17, 18, 22], atopy was assessed using only questionnaires. However, it was preferable to determine the serum level of total immunoglobulin E (IgE) [11, 12, 16] or of specific IgE [11] or of eosinophil [12] or to estimate sensitization to allergens with prick testing [12, 13].

There are few studies [11, 20] that assessed the reference equations of Fe_NO_ in healthy adults nonsmoking adults, as in the present study. As in most studies [9, 11, 13, 16–18, 22], smoking status was subjectively assessed via the medical questionnaire. It was preferable to objectively assess it, for example, via serum cotinine levels [15].

The present study noninclusion criteria were similar to those applied in similar studies [9–24]. Obesity was present in 26% of the total samples. The included group composition reflected a ‘‘healthy” population, since 28% of the general local population over 20 years showed obesity [46]. In addition, as did some other authors, adults having obesity were not excluded [9–13, 15–18, 22].

To our knowledge, the present study is the first that measured lung volumes, which are important data for the diagnosis of restrictive defect and lung hyperinflation [27, 47].

Detailed discussion of the subject's data appears in the Supplemental Data.

### 4.2. Fe_NO_ Measurement

As in some studies [17, 19, 24], an electrochemical analyzer (Medisoft) was used. The majority of studies concerning adult Fe_NO_ norms [9–16, 18, 20–23] have used the chemiluminescence analyzers.

Because environmental NO can reach high levels relative to those in exhaled breath, standardized techniques must prevent the contamination of biological samples with ambient NO [1]. As recommended [1] notwithstanding which technique is used, ambient NO at the time of each test should be recorded. In the present study, mean ± SD (minimum-maximum) ambient NO concentration was 1.4 ± 1.4 ppb (0–5 ppb). Medisoft device has an absorption column with high capacities for detecting and eliminating ambient NO. Thus its function is not limited by the values of ambient NO.

Because plethysmographic maneuvers transiently reduce the Fe_NO_ levels [1], NO analysis was performed before plethysmography.

As measurements need to be standardized for time of day (circadian rhythm effects [21]), Fe_NO_ measurements were performed in the same period of the day.

### 4.3. Statistical Analysis

The dependent variable (Fe_NO_) was logarithmically transformed in natural logarithm, as published elsewhere [10, 22].

The absolute values of Fe_NO_ were presented as geometric mean ± SD (95% CI, LLN, and ULN) and as minimum-maximum. In other studies [9–24], absolute values of Fe_NO_ were presented with great heterogeneity using several central tendency and dispersion measures (mean, geometric mean, median, interquartile range, SD, and 95% and 90% CIs) and in different subgroups.

Similar to other studies [11, 12], Fe_NO_ norms were presented in two ways: total sample reference equation and a table of limit values. In literature, Fe_NO_ norms were presented as reference equations [11, 12, 14, 16, 20–22, 24], as fixed threshold [9, 17], and as tables of limit values [10–13, 18, 19, 23].

Fe_NO_ reference norms should be further refined in the future [7], perhaps in ways similar to those recently reviewed for lung function measurements [48]. For example, samples with a wider range of ages and different races or ethnicities, multicentre research teams, and the use of standardized technical and statistical procedures are desirable features for Fe_NO_ norms studies [7].

### 4.4. Non-Disease-Related Subject Factors Influencing Fe_NO_ Values

Interpretation of  Fe_NO_ values relies upon comparison with predicted values available from published norms [9–24]. To our knowledge, the present study is the first that reported Fe_NO_ norms for healthy Arab Tunisian adults. Therefore, there is a continuing need for such clinical research.

The multivariate analysis showed that height significantly affects the Fe_NO_ data. In addition, females Fe_NO_ data were significantly affected by MEF_50_ and TGV. These factors will be analyzed in the following sections.

*Height Effect.* Like other studies [11, 14, 20, 21, 24] the present one reported an association between height and Fe_NO_. The origin in the airway epithelium indicates that the total surface area of the airway mucosa will be an important determinant for Fe_NO_ [7]. Indeed, the airway diffusing capacity for NO, which theoretically should be dependent on the airway mucosal surface area, has been shown to correlate with anatomic dead space volume in healthy subjects [49]. It is logical that height was found to be an important factor when evaluating Fe_NO_ values, as seen for other lung function parameters [27, 28, 31, 32]. 

*Lung Function Effect*. Although the influence of lung function has been described in few studies [22, 25], it was a significant predictor for Fe_NO_ in the present study. In fact, for the included females, MEF_50_ (%) and TGV (L) explained a slight but significant Fe_NO_ variability. This result is in in agreement with Liu et al. published norms [22], where FVC was included in the reference equation. These authors [22] have extensively described the relationship between lung function and Fe_NO_. 

*Why Does Pulmonary Function Influence* *Fe*_*NO*_ *[22]?* It has been demonstrated that Fe_NO_ levels may vary with the airway caliber [1], perhaps because of a mechanical effect on NO output. The percent values of MEF_50_, sensitive to the small airway, are more likely to present underlying lung disease. The absolute value of TGV, indicating lung size more rationally than a percentage of predicted values, is sensitive to lung hyperinflation and so for small airways [27]. Given that MEF_50_ correlated with Fe_NO_ only in females, one wonders whether females' small airways produced more NO. The hypothesis of flow dependence advanced by Liu et al. [22] cannot be retained, since FVC and FEV_1_ were not positively associated with Fe_NO_ values.

Conversely, sex, age, weight, BMI, BSA, and obesity status were not significantly associated with Fe_NO_ when other variables were controlled. In the published studies [9–24] the following significant influencing factors were found: race, sex, age, weight, BMI, household smoke exposure, and session exam.

### 4.5. Why Are not the Findings about the Fe_NO_ Determinants Consistent with Previous Literature?

Many explanations can be advanced especially about methodological factors and inclusion of additional significant influencing factors. 

*Methodological Factors.* The low percentage variance explained by the retained reference equation (*r* ^2^ = 2%) reveals the possible difficulty to determine the effect of different exogenous factors and their combination with Fe_NO_ [7]. For example, the effect of atopy cannot easily be captured in a single factor, because atopy may result in an increase in Fe_NO_ of anywhere between zero and several hundred ppb depending on the degree of IgE sensitization and the level of allergen exposure. However, this does not rule out the benefit of adjusting for the more predictive effect of, for example, age, height, and sex on expected normal Fe_NO_ values. Another source of variation of the reference equations published [9–24] may be the use of different Fe_NO_ analysers or calibration procedures [50] or the method of measure (Medisoft versus NIOX) [45], even though all studies reported that they were following the ATS/ERS guidelines [1, 51]. As Fe_NO_ values obtained with different devices are not directly comparable and may differ to a clinically relevant, as the device is used [44], the present data were adjusted according to Brooks et al. [45]. As can be seen (Figure 1) and even after adjustment (Supplemental Figure 3), the present study mean ± SD measured Fe_NO_ was significantly overestimated by the Saudi Arabian male reference equations [16]. Sample sizes, age groups, race-ethnic constituencies, and noninclusion criteria of reference populations in other studies [9–24] make it difficult to compare findings. Therefore, care must be taken when comparing the present study Fe_NO_ results with those using different machines in different studies [9–24]. Thus, the use of other studies Fe_NO_ norms may lead to misinterpretation of the Fe_NO_ values. The definition and future use of specific guidelines on how to report studies on reference values may contribute to the standardization of reports [7]. Published Fe_NO_ recommendations [1] are helpful in the standardization of the measurement, but not in the standardization, of how the methods are described [7]. 

*Inclusion of Additional Significant Influencing Factors.* Additional significant influencing factors were included in adults' Fe_NO_ norms [9–24]: race, ethnicity, atopy, allergy, total IgE, serum eosinophil cationic protein, smoking status, interaction between sex and smoking habits, asthma diagnosis, ambient NO, and upper respiratory tract infection symptoms. In addition, interindividual differences in NO synthase basal levels (e.g., variants in the neuronal NOS 1 gene [20, 21, 52]) can account for the missing variability. The effects of race and atopy are analyzed in the following sections and the other additional influencing factors are discussed in the Supplemental Data. 

*Race Effect.* Among the published studies [9–24], some have included non-Caucasian subjects such as African Americans [15], Arab [16], or Asian [17, 19–24]. The effect of race on Fe_NO_ values is now well established [21, 24] and current data are vastly adequate to allow conclusions about people of other genetic background [7, 21, 24]. There is evidence that race and ethnicity play an important role in lung function prediction [53]. 

*Atopy Effect.* For the definition of reference values for Fe_NO_, atopy is an essential variable and its assessment using questionnaire data is insufficient. There is good evidence that Fe_NO_ mainly reflects atopy in population studies [1, 54]. For that reason, the atopic status of study subjects was determined (using questionnaires) and only healthy nonatopic adults were included.

Jacinto et al. [7] have suggested that the methodology and reporting on normal Fe_NO_ values and the corresponding reference equations should be standardized and the formulation of reference equations should be based on a preset physiological model with endogenous and stable (at least in the short term) factors such as sex, age, and height. Furthermore, the influence of exogenous factors should be minimized in the population under study, for example, by using objective allergy testing and objective markers of exposure to cigarette smoke [7].

### 4.6. Fe_NO_ Norms and Interpretation

Among the published Fe_NO_ norms for adults [9–24], none have proposed a clear method of interpreting the measured Fe_NO_ or has provided a prospective verification of their studied populations.

Fe_NO_ values can be difficult to interpret, as they are strongly influenced by several intraindividual factors, including anthropometric data, sex, atopy, and smoking habits [7]. This is one of many problems with diagnostic tests, as recently discussed [48]: it is difficult to define “normality” in a given assessment [55]. Moreover, the numeric value of a diagnostic test can be presented in several forms: the absolute value and the percent predicted of a reference value.

The ATS guidelines [2] suggested that decision cut points rather than reference values be used when interpreting Fe_NO_ levels. Specifically, the guidelines stated that an adult Fe_NO_ < 25 ppb indicates a low likelihood of eosinophilic inflammation and corticosteroid response, whereas an adult Fe_NO_ > 50 ppb indicates otherwise. However, these cut points have not been validated in the Arab Tunisian population. At the heart of determining cut points is the definition of “normality,” which can be taken as representing 95% of the healthy general population [9, 21]. Based on this assumption, the present study showed that values exceeding 26 ppb for adults 18 to 60 years of age indicated abnormality and a high risk of airway inflammation. Coincidentally, the ATS threshold of 25 ppb for adults is very close to the maximum value observed in the Arab Tunisian population. The ATS upper threshold of 50 ppb for 18 to 60 years of age was 24 ppb above the maximum value and could possibly be lowered as proposed by See and Christiani [21].

Due to the inadequacy of the Saudi Arabian males' Fe_NO_ reference equations [16], norms adapted to Arab Tunisian population were established. For practical and routine interpretation of Fe_NO_, two ways were proposed: normal absolute values range taking into consideration age and height ranges and a reference equation taking height into account.

The interpretation of Fe_NO_ currently involves the use of absolute values reported in ppb, both in clinical practice and research, although absolute values are seldom used in respiratory medicine diagnostic tests [7]. According to the present study, we recommend the use of the total sample Fe_NO_ maximum value as a threshold, and each adult Fe_NO_ value higher than 26 ppb will be considered as abnormal. This method is much simpler for clinicians to remember and device manufacturers to program. In practice, it has been proposed that a “personal best” value for Fe_NO_ might be used [7, 56]. This is a strong approach if the objective is to monitor Fe_NO_. However, for the initial assessment of Fe_NO_ in a patient, this method is questionable [7]. Furthermore, the personal best values were shown to be close to published reference values [7, 56].

The percentage predicted of the reference value is now a standard transformation in most lung function laboratories [48]. Thus, their use to calculate reference values may be a practical and clinically useful approach [7]. Jacinto et al. [7] suggested the use of a similar approach when interpreting Fe_NO_ values using the percentage predicted of the reference value. A reference equation should include only easily measured anthropometric data that appear to influence Fe_NO_. For a practical interest, and as sex does not significantly affect the Fe_NO_ value, authors recommend the use of the total sample reference equation (Box 2) when calculating a predicted Fe_NO_ value. As recommended [40], the ULN to add to the predicted value was mentioned. The observed Fe_NO_ for each individual is then deemed to be abnormally high if it exceeds the ULN of the predicted mean [21]. This would allow clinicians to individualize decision making according to the unique characteristics of each person. To the best of our knowledge, the present study is the first to suggest a clear way to interpret a measured Fe_NO_ value. However, Leon de la Barra et al. [8] stated that correcting Fe_NO_ using reference equations did not enhance the performance characteristics of Fe_NO_ as a predictor of either the diagnosis of asthma or steroid responsiveness in patients with chronic airways related symptoms.

Further research is needed to clarify the Fe_NO_ method of interpretation [7]. Nevertheless, the individual factors taken into consideration will be an important step to improve the interpretation of Fe_NO_ values [7]. Such factors are easily accessible at the clinic and incorporating them will require very little extra effort [7]. Most importantly, if reference equations are used, clinical cut-offs can be generalized across age groups and genetic backgrounds [7].

### 4.7. Reliability of the Local Fe_NO_ Norms

The reliability of the retained norms was confirmed in the prospectively studied population, confirming the continuing need of establishing regional reference norms [1]. This argues for the use of specific reference norms in the Arab Tunisian population. The implications of this for adults with bronchial asthma may be considerable, resulting in a false-positive misdiagnosis of bronchial inflammation.

In conclusion, reliable norms to interpret the results of Fe_NO_ were established in healthy Tunisian Arab adults. The Fe_NO_ can easily be predicted according to a reference equation taking into consideration height or age and height table ranges. Local Fe_NO_ norms enrich the World Bank of Fe_NO_ norms the clinician can use to choose the most appropriate norms based on an adult's location or ethnic group.

## Supplementary Material

The supplementary Material contains the following sections. The section Methods includes detailed information about the study design (especially recruitment method), and the published Fe_NO_ norms for Saudi Arabian males. The section Results contains four figures (distribution of the healthy total sample by sex and age range, measured Fe_NO_ in subgroups of healthy adults', according to age, height and weight ranges; comparison, for the same age range, of measured and predicted (after adjustment values) Fe_NO_ determined from Saudi Arabian norms; and measured Fe_NO_ values of the equation and validation groups according to height). It also includes four tables (Fe_NO_ norms for Arab populations; description of the applied non-inclusion criteria; healthy adults' plethysmographic data and comparison between the equation and validation groups' data). The section Discussion includes discussion of subject's data, inclusion of additional significant Fe_NO_ influencing factors and an attempt to answer the following question: what do “abnormal” Fe_NO_ values reflect?

## Figures and Tables

**Box 1:**
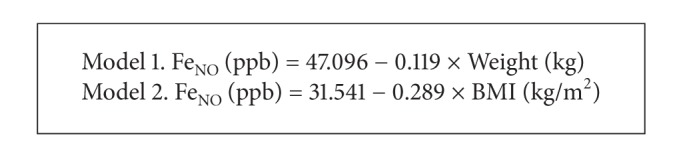
Fe_NO_ norms for Saudi Arabian males [16].

**Box 2:**
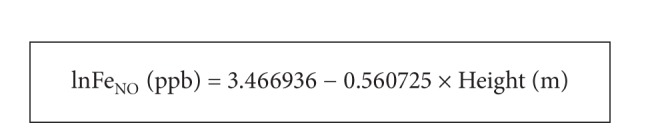
Retained Fe_NO_ reference equation.

**Table 1:** Healthy Arab Tunisian never-smoking adults' characteristics.

		Females (*n* = 145)	Males (*n* = 112)	Total sample (*n* = 257)
Anthropometric data (data are mean ± SD)				
Age	(year)	38.04 ± 11.55	41.09 ± 11.43*	39.37 ± 11.58
Weight	(kg)	70 ± 13	79 ± 14*	74 ± 14
Height	(m)	1.60 ± 0.06	1.73 ± 0.07*	1.65 ± 0.09
Body mass index	(kg·m^−2^)	27 ± 5	26 ± 4	27 ± 5
Body surface area	(m^2^)	1.72 ± 0.15	1.92 ± 0.18*	1.81 ± 0.19

Plethysmographic and fraction-of-exhaled-nitric-oxide (Fe_NO_) data (data are mean ± SD)				
FVC	(%)	99 ± 12	95 ± 11*	97 ± 12
FEV_1_	(%)	96 ± 11	93 ± 11*	94 ± 11
PEF	(%)	78 ± 14	79 ± 14	79 ± 14
MMEF	(%)	89 ± 21	82 ± 18*	86 ± 20
MEF_25_	(%)	74 ± 29	73 ± 34	74 ± 31
MEF_50_	(%)	85 ± 20	87 ± 20	86 ± 20
MEF_75_	(%)	82 ± 15	86 ± 16	84 ± 16
TLC	(%)	96 ± 11	92 ± 12*	94 ± 12
TGV	(%)	98 ± 20	105 ± 28*	101 ± 24
RV	(%)	101 ± 32	101 ± 37	101 ± 34
Fe_NO_	(ppb)	13.31 ± 4.55	13.84 ± 5.26	13.54 ± 4.87
lnFe_NO_	(ppb)	2.53 ± 0.35	2.55 ± 0.40	2.54 ± 0.37

Obesity status (data are number (%))				
	Normal weight	51 (35%)	44 (39%)	95 (37%)
Obesity status	Overweight	49 (34%)	47 (42%)	96 (37%)
	Obesity	45 (31%)	21 (19%)**	66 (26%)

For abbreviations, ln: natural logarithm.

Plethysmographic data are expressed as percentage (%) of predicted value.

*P < 0.05 (Mann-Whitney *U*-test): females versus males.

**P < 0.05 (chi-2): females versus males.

**Table 2:** Univariate analysis between the fraction-of-exhaled-nitric-oxide (Fe_NO_) and healthy Arab Tunisian never-smoking adults' data.

		Females (*n* = 145)	Males (*n* = 112)	Total sample (*n* = 257)
Univariate analysis between Fe_NO_ data and continuous measures				
Age	(yr)	0.08	0.01	0.05
Weight	(kg)	−0.04	0.04	0.02
Height	(m)	−0.30*	−0.18*	−0.13*
Body mass index	(kg·m^−2^)	0.08	0.14	0.10
Body surface area	(m^2^)	−0.13	−0.02	−0.03
FVC	(L)	−0.23*	−0.09	−0.07
FVC	(%)	−0.03	0.01	−0.02
FEV_1_	(L)	−0.24*	−0.09	−0.09
FEV_1_	(%)	−0.06	−0.01	−0.04
FEV_1_/FVC	(Absolute value)	−0.09	−0.01	−0.06
PEF	(L/s)	−0.22*	0.08	−0.01
PEF	(%)	−0.14	0.13	−0.01
MMEF	(L/s)	−0.21*	−0.04	−0.10
MMEF	(%)	−0.18*	−0.02	−0.12
MEF_25_	(L/s)	−0.17*	−0.03	−0.09
MEF_25_	(%)	−0.13	0.02	−0.05
MEF_50_	(L/s)	−0.23*	−0.06	−0.11
MEF_50_	(%)	−0.19*	−0.03	−0.11
MEF_75_	(L/s)	−0.22*	0.06	−0.01
MEF_75_	(%)	−0.17*	0.11	−0.02
TLC	(L)	−0.19*	−0.16	−0.08
TLC	(%)	−0.01	−0.09	−0.06
TVG	(L)	−0.20*	−0.15	−0.11
TVG	(%)	−0.14	−0.11	−0.12*
RV	(L)	−0.07	−0.17	−0.10
RV	(%)	−0.03	−0.15	−0.09

Univariate analysis between Fe_NO_ data and obesity status				
Obesity status	Normal weight or overweight	13.02 ± 4.52	13.87 ± 5.15	13.42 ± 4.84
	Obesity	13.96 ± 4.58	13.71 ± 5.87	13.88 ± 4.98

For abbreviations, see abbreviations list.

*P < 0.05 (univariate Spearman correlation coefficients between Fe_NO_ data and continuous measures).

**P < 0.05 (*t*-tests): females versus males.

**Table 3:** Independent variables included in the forward linear stepwise multiple regression model for the natural logarithm of fraction-of-exhaled-nitric-oxide (Fe_NO_).

Independent variables	Nonstandardized regression coefficient (*B*)	95% confidence interval around each *B*	Cumulative determination coefficient (*r* ^2^)	*P* level	Standard error	1.64 residual standard deviation
Females (*n* = 145)						
Constant	4.73424	3.48017 to 5.98832		0.000000		0.54553
Height (m)	−1.17043	−2.00733 to −0.33353	0.0685	0.023293	0.7534	
MEF_50_ (%)	−0.00209	−0.00446 to 0.00028	0.0839	0.149905	0.7510	
TGV (L)	−0.06149	−0.14921 to 0.02623	**0.0924**	0.252243	0.7647	

Males (*n* = 112)						
Constant	4.47053	2.95938 to 5.98168		0.000004		0.641486
Height (m)	−1.11099	−1.98559 to −0.23639	**0.0380**	0.039545	0.9214	

Total sample (*n* = 257)						
Constant	3.466936	2.78519 to 4.14868		0.000000		0.5992724
Height (m)	−0.560725	−0.97258 to −0.14887	**0.0192**	0.026431	0.41569	

For abbreviations, see abbreviations list.

For females: lnFe_NO_ (ppb) = 4.73424 − 1.17043 × height (m) − 0.00209 × MEF_50_ (%) − 0.06149 × TGV (L).

For males: lnFe_NO_ (ppb) = 4.47053 − 1.11099 × height (m).

For the total sample: lnFe_NO_ (ppb) = 3.466936 − 0.560725 × height (m).

**Table 4:** Fraction-of-exhaled-nitric-oxide (Fe_NO_) norms: Fe_NO_ data (ppb) according to ranges of height and age among 257 healthy Arab Tunisian never-smoking adults.

Height ranges (m)	Age ranges (year)				
	[17–35[	[35–45[	[45–55[	≥55	All ranges of age
**[1.36–1.55[**	16 ± 6 [9–**22**] (*n* = 5)	15 ± 5 [5–**24**] (*n* = 10)	15 ± 3 [12–**20**] (*n* = 8)	13 ± 6 [7–**21**] (*n* = 4)	15 ± 5 [5–**24**] (*n* = 27)
** [1.55–1.65[**	12 ± 5 [5–**24**] (*n* = 41)	13 ± 5 [7–**26**] (*n* = 29)	14 ± 4 [8–**22**] (*n* = 25)	11 ± 5 [6–**23**] (*n* = 8)	13 ± 5 [5–**26**] (*n* = 103)
** [1.65–1.75[**	12 ± 3 [7–**21**] (*n* = 27)	13 ± 6 [7–**25**] (*n* = 27)	15 ± 5 [8–**25**] (*n* = 18)	11 ± 7 [7–**19**] (*n* = 9)	13 ± 5 [7–**25**] (*n* = 81)
**≥1.75**	12 ± 5 [5–**23**] (*n* = 17)	14 ± 6 [6–**24**] (*n* = 10)	11 ± 5 [6–**21**] (*n* = 17)	12 ± 2 [11–**14**] (*n* = 2)	12 ± 5 [5–**24**] (*n* = 46)

All ranges of height	12 ± 4 [5–**24**] (*n* = 90)	13 ± 5 [5–**26**] (*n* = 76)	13 ± 5 [6–**25**] (*n* = 68)	11 ± 5 [6–**23**] (*n* = 23)	13 ± 5 [5–**26**] (*n* = 257)

Data are geometric mean ± standard deviation [minimum–maximum].

*n* = number of adults in each range.

Algorithm of interpretation:

*Step  1*. Determine each adult age and height ranges.

*Step  2*. Note, for these ranges, the Fe_NO_ maximum value (values in bold character).

*Step  3*. The measured Fe_NO_ is considered as abnormal when it is higher than the predicted Fe_NO_ maximum value previously determined.

## Abbreviation List

ATS: American Thoracic Society
BMI: Body mass index
BSA: Body surface area
ERS: European Respiratory Society
Fe_NO_: Fraction of nitric oxide in exhaled breath
FEV1: 1st second forced expiratory volume
FVC: Forced vital capacity
IgE: Immunoglobulin E
LLN: Lower limit of normal
ln: Natural logarithm
MEF*x*%: Forced expiratory flow when *x*% of FVC has been exhaled
MMEF: Maximal mid expiratory flow
PEF: Peak expiratory flow
ppb: Parts per billion
*r*: Correlation coefficient
*r*^2^: Determination coefficient
RSD: Residual standard deviation
RV: Residual volume
SD: Standard deviation
TGV: Thoracic gas volume
TLC: Total lung capacity
ULN: Upper limit of normal
95% CI: 95% confidence interval.
