# Supplementary material for: Fraction of Exhaled Nitric Oxide (FeNO) Norms in Healthy Tunisian Adults
Source: Biomed Res Int. 2014 Jun 3;2014:269670. doi: 10.1155/2014/269670 (PMC4065671; doi:10.1155/2014/269670)
Supplement: Supplementary file 1 — The supplementary Material contains the following sections. The section Methods includes detailed information about the study design (especially recruitment method), and the published FeNO norms for Saudi Arabian males. The section Results contains four figures (distribution of the healthy total sample by sex and age range, measured FeNO in subgroups of healthy adults', according to age, height and weight ranges; comparison, for the same age range, of measured and predicted (after adjustment values) FeNO determined from Saudi Arabian norms; and measured FeNO values of the equation and validation groups according to height). It also includes four tables (FeNO norms for Arab populations; description of the applied non-inclusion criteria; healthy adults' plethysmographic data and comparison between the equation and validation groups' data). The section Discussion includes discussion of subject's data, inclusion of additional significant FeNO influencing factors and an attempt to answer the following question: what do “abnormal” FeNO values reflect? [file 269670.f1.zip › 269670.f1/Supplementary_Data_R1_2738_911454.docx]

**supplementaRY DATA**

**Title.** Fraction of Exhaled Nitric Oxide (Fe_NO_) Norms in Healthy Tunisian Adults

**Authors.** Sonia ROUATBI, Mohamed Ali CHOUCHENE, Ines SFAXI, Mohamed BEN REJEB, Zouhair TABKA, Helmi BEN SAAD.

***POPULATION AND METHODS***

***Study design***

Subjects were recruited from the Hospital workers, the parents of Medical school students and the public. Informational letters clarifying the aims of the study were put up in the Hospital departments and the school of Medicine. When an adult was interested, an appointment for a medical questionnaire and exploration was fixed. Data from each volunteer adult included: sex, age, height, weight, smoking history (active or passive), medication use, medical history, physical examination, Fe_NO_ and plethysmography data. All adults received a copy of their exploration, and when an unknown dysfunction was discovered, they were sent to a specialist.

***Statistical analysis***

Published *Fe_NO_ norms for Saudi Arabian males*[**^1^**](#_ENREF_1) is extensively detailed in the ***Supplementary Table I***.

The model of the natural logarithm linear regression equation is the following: LnFe_NO_ (ppb) = B_1_ x categorical variable_1_ + B_2_ x categorical variable_2_ + B_n_ x categorical variable_n_ + Bx_1_ x continuous variable_1_ + Bx_2_ x continuous variable_2_ + Bx_n_ x continuous variable_n_ + Constant. B is the non-standardized regression coefficient. 95% CI around each B was determined.

***RESULTS***

**Non-inclusion criteria**

Non-inclusion criteria are presented in the **Supplemantary Table II**.

From an initial sample of 400 voluntary adults, non-inclusion criteria were found in 93 subjects. The remaining 307 adults were divided into two groups: **equation** (145 females and 112 males) and **validation** (25 females and 25 males) **groups’**.

***Subject’s data***

**Supplementary Table 3** exposes the total sample plethysmographic data expressed in absolute values.

**DISCUSSION**

***Subject’s data***

As for almost all the studies aiming to publish Fe_NO_ norms[**^1-15^**](#_ENREF_1), the present study was not a random population sample. Some caution should be warranted when interpreting the results of cross-sectional studies in volunteers, because of a possible selection bias and cohort effects[**^16^**](#_ENREF_16). Thus, longitudinal studies analyzed by appropriate statistical models are necessary to correctly describe the functional changes associated with age[**^17^**](#_ENREF_17).

The present study sample size (n=257) was higher than in some studies [n=106[**^10^**](#_ENREF_10), n=121[**^1^**](#_ENREF_1), n=122[**^4^**](#_ENREF_4), n=166[^12^](#_ENREF_12), n=193[**^2^**](#_ENREF_2), n=200[^15^](#_ENREF_15), n=204[**^3^**](#_ENREF_3), n=240[**^9^**](#_ENREF_9), n=249[**^8^**](#_ENREF_8)] but was smaller than in some previous studies [n=895[**^5^**](#_ENREF_5)**^,^**[**^7^**](#_ENREF_7), n=897[**^11^**](#_ENREF_11), n=1093[**^13^**](#_ENREF_13), n=1131[**^6^**](#_ENREF_6)]. It is important to note that the subgroup aged > 55 years with a height > 1.75 m includes only two subjects and the data obtained in this subgroup cannot be extrapoled to the general population.

The validation group number (n=50) seems small, but it is sufficient to provide adequate validation since it is closer to that included in similar studies aiming to validate Tunisian reference equations for children Fe_NO_ data and adults 6-min walk distance data, respectively, (n=24[**^18^**](#_ENREF_18) and n=30[**^19^**](#_ENREF_19)).

***Inclusion of additional significant influencing factors***

Additional significantly influencing factors were included in the published adults’ Fe_NO_ norms[**^1-15^**](#_ENREF_1)**^,^**[**^20^**](#_ENREF_20): race, ethnicity, atopy, allergy, total IgE, serum eosinophil cationic protein, smoking status, interaction between sex and smoking habits, asthma diagnosis, ambient NO and upper respiratory tract infection symptoms.

Like most of the published studies[**^1-6^**](#_ENREF_1)**^,^**[**^8-10^**](#_ENREF_8), smoking habits were not assessed with objective methods. For the definition of reference values for Fe_NO_, smoking status is an essential variable and its assessment using only questionnaire data seems insufficient. Chronically reduced levels of Fe_NO_ have been demonstrated in cigarette smokers in addition to acute effects immediately after cigarette smoking[**^21^**](#_ENREF_21)**^,^**[**^22^**](#_ENREF_22). Despite the depressing effect of smoking, smokers with asthma still have a raised Fe_NO_[**^23^**](#_ENREF_23). For that reason, the ATS/ERS recommend that subjects should not smoke in the hour before measurements, and short- and long-term active and passive smoking history should be recorded[**^21^**](#_ENREF_21).

In addition to individual-specific factors, several behavioural and environmental factors have been pointed out as influencing Fe_NO_[**^24^**](#_ENREF_24), such as rhinovirus infections[**^7^**](#_ENREF_7), allergen exposure[**^25^**](#_ENREF_25), physical exercise[**^26^**](#_ENREF_26), ozone exposure[**^27^**](#_ENREF_27), air pollution[**^28^**](#_ENREF_28) and nutritional history[**^16^**](#_ENREF_16).

**What do “abnormal” Fe_NO_ values reflect?**

The use of Fe_NO_ in clinical practice has been the focus of numerous studies and considerable debate[**^29^**](#_ENREF_29)**^,^**[**^30^**](#_ENREF_30). Vijverberg et al.[^30^](#_ENREF_30) concluded that High Fe_NO_ is a poor marker of asthma control in children with reported use of asthma medication. Leon de la Barra et al.[**^29^**](#_ENREF_29) stated that “there is an emerging consensus, poised somewhere between outright enthusiasm and sceptical nihilism, that the utility of Fe_NO_ is greatest in identifying the potential (or not) for steroid responsiveness and in assessing complex asthma in which there is significant discordance between the symptoms which the patient is reporting, and the intensity of underlying airway inflammation”. These two aspects were simultaneously considered by Perez de Llano et al.[**^31^**](#_ENREF_31). The following question was asked: can Fe_NO_ be used to predict patients with poorly controlled asthma, in whom additional inhaled or oral steroid would or would not be helpful? Interestingly, almost 50% did not gain control despite maximum doses of inhaled fluticasone and the addition of oral steroid[**^31^**](#_ENREF_31). Fe_NO_ values of less than 30 ppb were associated with predictive values of >90% for the absence of response to high dose therapy. These results demonstrate the usefulness of Fe_NO_ in directing the clinician away from inappropriate treatment.

Some studies have demonstrated that Fe_NO_ measurement might be useful for the diagnosis of asthma in adults[**^32^**](#_ENREF_32)**^,^**[**^33^**](#_ENREF_33)*.* However, in some obese asthmatic patients, very low Fe_NO_ levels were observed[**^34^**](#_ENREF_34). In fact it has been shown in asthma patients that BMI and plasma ratio of leptin/adiponectin is associated with reduced Fe_NO_ and that BMI is associated with increased exhaled 8-isoprostanes[**^34^**](#_ENREF_34). In addition, Fe_NO_ measurement has also been investigated in the treatment algorithm for asthma. However, randomised controlled algorithm asthma control trials revealed equivocal benefits when adding Fe_NO_ measurement to the routine guideline management including spirometry[**^35^**](#_ENREF_35)**^,^**[**^36^**](#_ENREF_36).

**SUPPLEMENTARY FIGURES LEGENDS**

**Supplementary Figure 1.** Distribution of the Tunisian healthy total sample by sex and age range.

***p < 0.05:** females vs. males.

**Supplementary Figure 2.** The measured fraction-of-exhaled-nitric oxide (Fe_NO_) in subgroups of 257 healthy Tunisian adults’, according to age (**FigA**), height (**FigB**) and weight (**FigC**) ranges. n=number of adults. Data are shown as box-and-whiskers-plots illustrating the mean (**􀀀**), standard error ( ) and 95% confidence interval ( ).

***p<0.05:** Comparison (T-test) from one range to the next.

**NS:** not significant.

**Supplementary Figure 3.** Comparison, for the same age range, of measured and predicted (after adjustment according to Brooks et al.) fraction-of-exhaled-nitric-oxide (Fe_NO_) determined from Saudi Arabian norms:

**Fig 3A.** Model including weight.

**Fig** **3B.** Model including body mass index.

n=number of males having the age range of the Saudi Arabian predicted Fe_NO_ study.

Solid line ( ): regression line. Dashed line ( ): identity line. **r^2^:** coefficient of determination. **r:** correlation coefficient. **p**: probability

**Supplementary Figure 4.** The measured fraction-of-exhaled-nitric-oxide (Fe_NO_) values of the equation and validation groups according to height.

**Ο**: Males of the Equation group (n=112)

⬤: Males of the Validation group (n=25)

□: Females of the Equation group (n=145)

⏹: Females of the Validation group (n=25)

| **Supplementary Table 1. Fraction-of-exhaled-nitric-oxide (Fe_NO_) norms for Arab populations.** | | |
| --- | --- | --- |
|  | **Kingdom of Saudi Arabia: Habib et al.**[**^1^**](#_ENREF_1) | **Tunisia**  **(present study)** |
| **Period of study** | September 2007- August 2008 | May 2012- December 2012 |
| **Time of**  **Fe_NO_ test** | 9 a.m to 11 a.m | 8 a.m to 12 a.m |
| **Recruitment method** | Medical students and hospital personnel | Convenience sample: medical students and hospital personnel |
| **Non-inclusion criteria** | Smoking, recent/Current upper RTI, medications, atopy, clinical manifestations of allergic diseases, abnormal spirometry, abnormal serum IgE levels, respiratory disease, symptoms of respiratory disease (last 1 year) | Hay fever, chronic illnesses, oto-rhino-laryngolocic diseases or symptoms, clinical manifestation of allergic diseases, pulmonary diseases or related respiratory symptoms, abnormal lung function, pregnancy, regular medication use, current or ex-smokers, inability to perform properly respiratory measurements. |
| **Precautions** | Refrain from eating, drinking and strenuous exercise 2 h before | Avoid eating (2 h), drinking (2 h), ingesting caffeine (2 h), strenuous activity (2 h) |
| **Guidelines** | ATS/ERS_2005_[**^21^**](#_ENREF_21) | ATS/ERS_2005_[**^21^**](#_ENREF_21) |
| **Material** | Chemiluminescence analyzer (NOX EVA 4000, Seres, aix-en-provence, France)  *Calibration* before each test | Electrochemical analyzer (Medisoft, Sorinnes [Dinant], Belgium)  *Calibration* before each test |
| **Technique** | Online | Online |
| **Flow** **(ml/s)** | 50±10% | 50 |
| **Other explorations** | Questionnaire, spirometry, total IgE | Questionnaire, plethysmography |
| **Individual Fe_NO_** | Mean of 3 exhalations  within 10% deviation | Mean of 3 exhalations  within 10% deviation |
| **Race** | Arab | Arab |
| **Sex** | M (n=121) | M (n=112); F (n=145) |
| **Age (Yr)** | 31.00±12.24**^*^**  19-64**^**^** | 39±12**^*^**  38 to 41**^***^** |
| **Height (m)** | 1.72±0.08**^*^**  1.49-1.88**^**^** | 1.65±0.09**^*^**  1.64 to 1.66**^***^** |
| **Fe_NO_ (ppb)** | 22.8±8.1**^*^**  7.7-46.6**^**^** | 13.54±4.87**^*^**  12.94 to 14.14**^***^** |
| **Fe_NO_ influencing factors** | W, BMI | H |
| **Fe_NO_ reference equation** | **Model 1.** Fe_NO_=47.096 - 0.119xW (kg)  **Model 2.** Fe_NO_=31.541- 0.289xBMI (kg/m^2^). | **Male:** LnFe_NO_ (ppb) = 4.470-1.111xH (m).  **Female:** LnFe_NO_ (ppb) = 4.734-1.170xH (m)-0.002xMEF_50_ (%)-0.061xTGV (L)  **Total sample:** LnFe_NO_ (ppb) = 3.467-0.561xH (m)  Any Fe_NO_ value greater than 26.00 ppb may be considered abnormal. |
| **r^2^ and RSD** | NR | **M:** r^2^=3.80%. RSD=0.390.  **F:** r^2^=9.24%. RSD=0.332.  **TS:** r^2^=1.92%. RSD=0.365. |
| **Interpretation method** | 84% of subjects:  Fe_NO_ < 30 ppb  95% of subjects:  Fe_NO_ < 40 ppb | Upper limit of normal |
| **Validation group** | No | Yes |
| **Comparison groups** | No | No |
| **Comparison with other studies** | No | Yes |
| **ATS:** American thoracic society. **BMI:** body mass index. **ERS:** European respiratory society. **F:** female. **H:** height. **h:** hour. **IgE:** immunoglobulin E. **Ln:** natural logarithm. **M:** male. **MEF_x_:** forced expiratory flow when x% of FVC has been exhaled. **n:** number. **NR:** not reported. **r^2^:** coefficient determination. **RSD:** residual standard deviation. **RTI**: respiratory tract infection. **TGV:** thoracic gas volume. **TS:** total sample. **W:** weight  **^*^:** data are mean±SD. **^**^:** data are minimum-maximum. **^***^**: data are 95% confidence interval. | | |

| Supplementary Table 2. Applied non-inclusion criteria (n=400) |  |
| --- | --- |
| **Brief description** | **Number** |
| Recent airway infection (cold, flu, sore throat within the last 7 days) | **21** |
| Current or ex-smokers | **18** |
| Poor cooperation to achieve a standard spirometry or a correct fraction-of-exhaled-nitric-oxide measure | **15** |
| Atopic history (allergic rhinitis, urticaria, recurrent symptoms of rhinitis, skin allergy) | **14** |
| Asthma or obstructive ventilatory defect | **12** |
| Medication use | **7** |
| Eating or/and drinking (water, caffeine) before measurement | **6** |

| **Supplementary Table 3. Healthy Arab Tunisian never-smoking adults’ plethysmographic data expressed in absolute values.** | | | | |
| --- | --- | --- | --- | --- |
|  | | **Females (n=145)** | **Males (n=112)** | **Total sample (n=257)** |
| FVC | (L) | 3.14±0.51 | 4.30±0.69**^*^** | 3.65±0.83 |
| FEV_1_ | (L) | 2.63±0.47 | 3.47±0.60**^*^** | 2.99±0.67 |
| FEV_1_/FVC | (absolute value) | 0.83±0.06 | 0.81±0.06**^*^** | 0.82±0.06 |
| PEF | (L/s) | 5.08±1.02 | 7.11±1.27**^*^** | 5.97±1.52 |
| MMEF | (L/s) | 2.93±0.86 | 3.53±1.00**^*^** | 3.19±0.97 |
| MEF_25_ | (L/s) | 1.39±0.66 | 1.57±0.84 | 1.47±0.75) |
| MEF_50_ | (L/s) | 3.52±0.92 | 4.30±1.16**^*^** | 3.86±1.10 |
| MEF_75_ | (L/s) | 4.77±0.99 | 6.64±1.26**^*^** | 5.58±1.45 |
| TLC | (L) | 4.53±0.64 | 6.16±1.05**^*^** | 5.24±1.17 |
| TGV | (L) | 2.56±0.56 | 3.48±0.98**^*^** | 2.96±0.89 |
| RV | (L) | 1.50±0.48 | 1.96±0.76**^*^** | 1.70±0.66 |
| For abbreviations, see abbreviations list. **^*^**p < 0.05 (Mann-Whitney U test): females vs. males. | | | | |

| **Supplementary Table 4. Equation and validation groups’ data.** | | | | |
| --- | --- | --- | --- | --- |
|  | | | **Equation group (n=257)** | **Validation group (n=50)** |
| **Anthropometric data (data are mean±SD)** | | | | |
| Sex | (Male/female) | | 112/145 | 25/25 |
| Age | (year) | | 39.37±11.58 | 40.88±13.45 |
| Weight | (kg) | | 74±14 | 75±22 |
| Height | (m) | | 1.65±0.09 | 1.66±0.09 |
| Body mass index | (kg.m^-2^) | | 27±5 | 28±9 |
| Body surface area | (m^2^) | | 1.81±0.19 | 1.82±0.24 |
| **Plethysmographic and fraction-of-exhaled-nitric-oxide (Fe_NO_) data (data are mean±SD))** | | | | |
| FVC | | (%) | 97±12 | 93±14**^*^** |
| FEV_1_ | | (%) | 94±11 | 91±12**^*^** |
| PEF | | (%) | 79±14 | 79±14 |
| MMEF | | (%) | 86±20 | 83±22 |
| MEF_25_ | | (%) | 74±31 | 77±39 |
| MEF_50_ | | (%) | 86±20 | 81±17 |
| MEF_75_ | | (%) | 84±16 | 83±17 |
| TLC | | (%) | 94±12 | 93±14 |
| TGV | | (%) | 101±24 | 105±32 |
| RV | | (%) | 101±34 | 116±44**^*^** |
| Fe_NO_ | | (ppb) | 13.54±4.87 | 12.98±4.83 |
| **Obesity status (data are number (%))** | | | | |
| Obesity status | | Normal weight | 95 (37%) | 18 (36%) |
|  |  | Overweight | 96 (37%) | 15 (30%) |
|  |  | Obesity | 66 (26%) | 17 (34%) |
| For abbreviations, see abbreviations list. Plethysmographic data are expressed as a percentage predicted value (%).  **^*^**p < 0.05 (Mann-Whitney U test): Equation group vs. Validation group.  **^**^**p <0.05 (chi-2): Equation group vs. Validation group. | | | | |

**Supplementary Figure 1.**

**
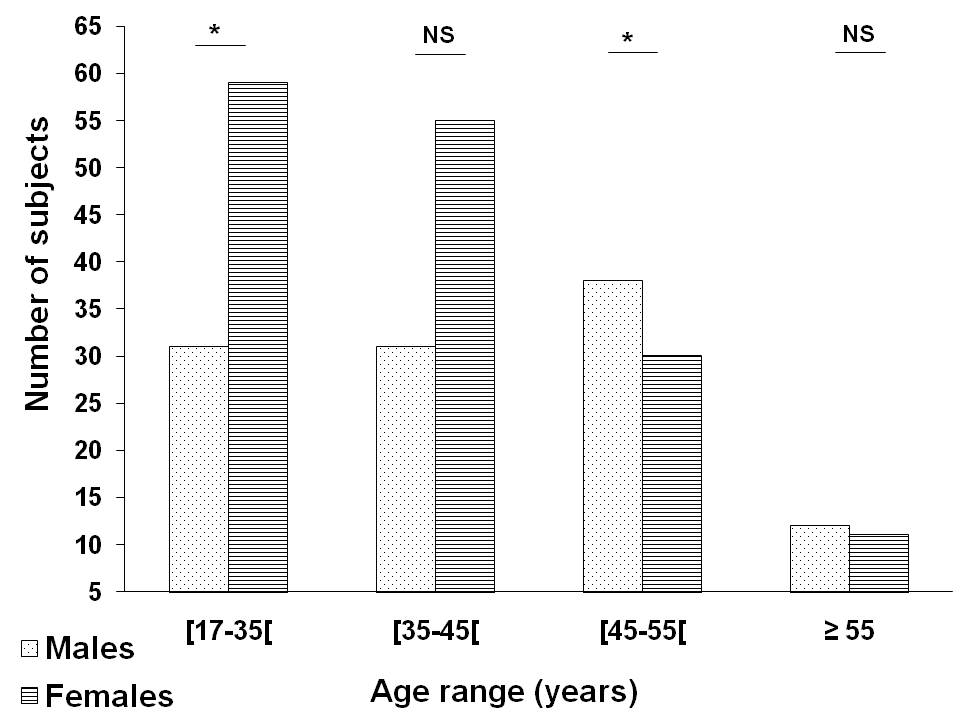
Supplementary Figure 2.**

**
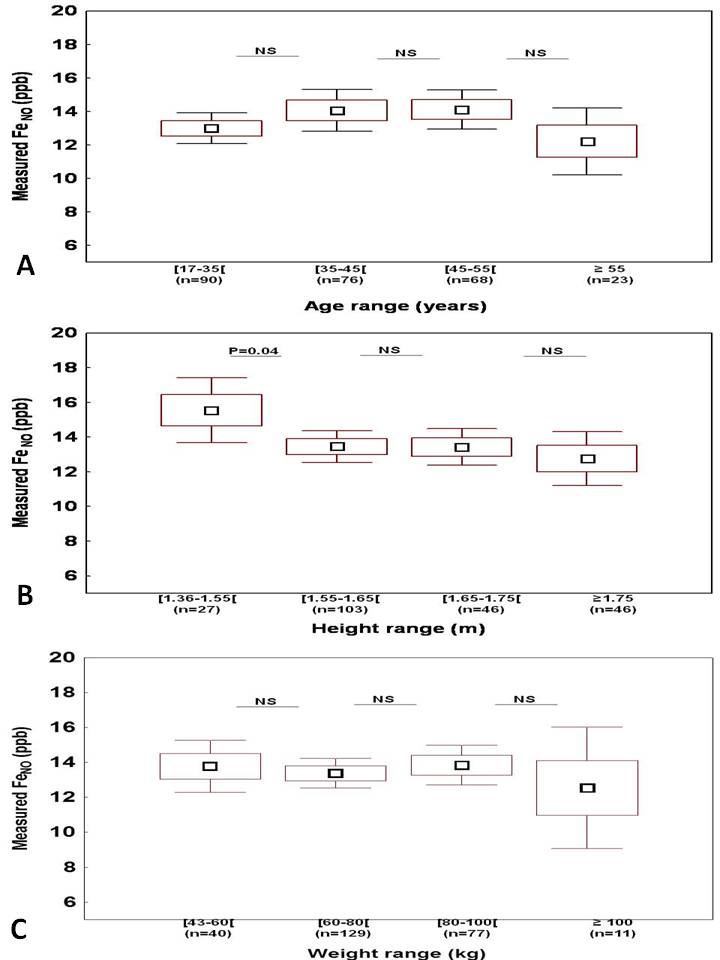
**

**Supplementary Figure 3.**

**
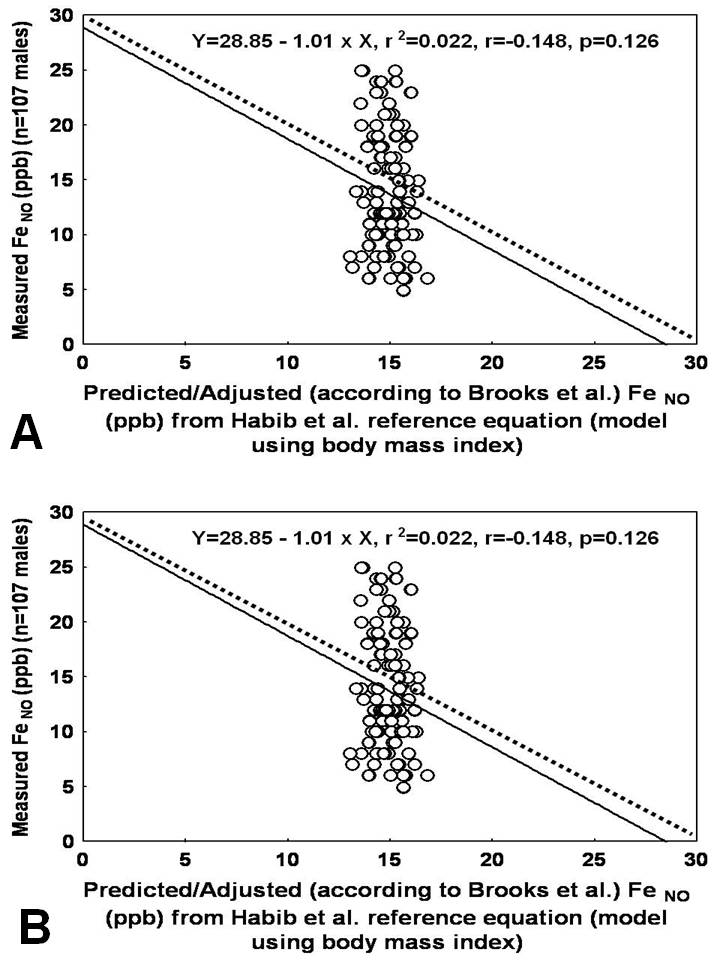
**

**Supplementary Figure 4.**

**
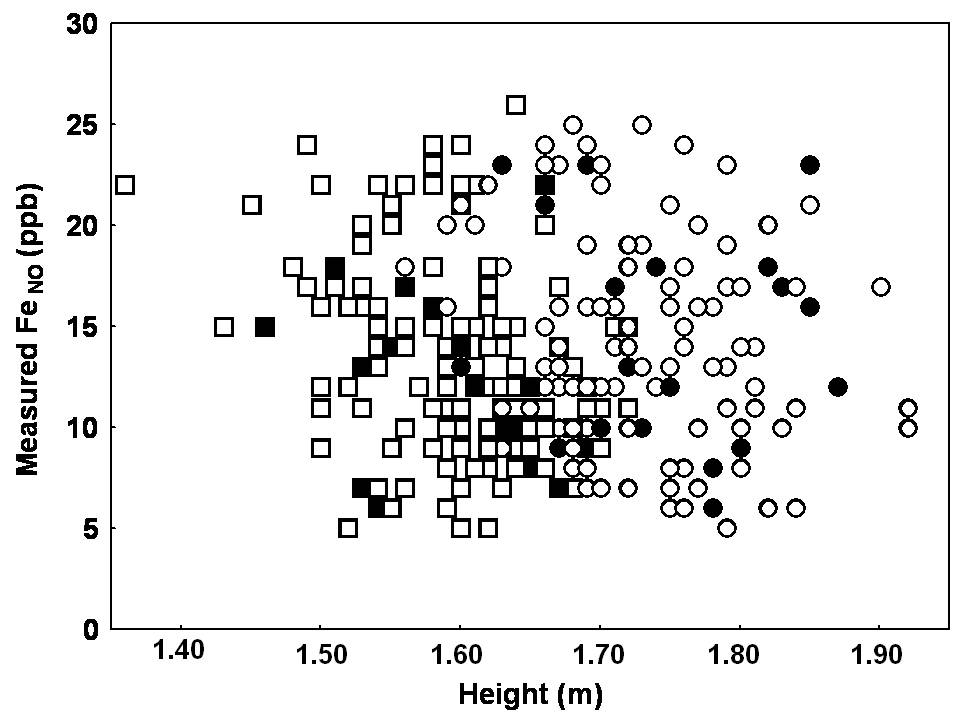
**

**REFERENCES**

1 Habib SS, Abba AA, Al-Zoghaibi MA, et al. Reference range values of fractional exhaled nitric oxide in healthy Arab adult males. Saudi Med J 2009; 30:1395-1400

2 Travers J, Marsh S, Aldington S, et al. Reference ranges for exhaled nitric oxide derived from a random community survey of adults. Am J Respir Crit Care Med 2007; 176:238-242

3 Olivieri M, Talamini G, Corradi M, et al. Reference values for exhaled nitric oxide (reveno) study. Respir Res 2006; 7:94

4 Maestrelli P, Ferrazzoni S, Visentin A, et al. Measurement of exhaled nitric oxide in healthy adults. Sarcoidosis Vasc Diffuse Lung Dis 2007; 24:65-69

5 Taylor DR, Mandhane P, Greene JM, et al. Factors affecting exhaled nitric oxide measurements: the effect of sex. Respir Res 2007; 8:82

6 Olin AC, Bake B, Toren K. Fraction of exhaled nitric oxide at 50 mL/s: reference values for adult lifelong never-smokers. Chest 2007; 131:1852-1856

7 Levesque MC, Hauswirth DW, Mervin-Blake S, et al. Determinants of exhaled nitric oxide levels in healthy, nonsmoking African American adults. J Allergy Clin Immunol 2008; 121:396-402 e393

8 Liu HC, Hsu JY, Cheng YW, et al. Exhaled nitric oxide in a Taiwanese population: age and lung function as predicting factors. J Formos Med Assoc 2009; 108:772-777

9 Matsunaga K, Hirano T, Kawayama T, et al. Reference ranges for exhaled nitric oxide fraction in healthy Japanese adult population. Allergol Int 2010; 59:363-367

10 Gelb AF, George SC, Camacho F, et al. Increased nitric oxide concentrations in the small airway of older normal subjects. Chest 2011; 139:368-375

11 Dressel H, de la Motte D, Reichert J, et al. Exhaled nitric oxide: independent effects of atopy, smoking, respiratory tract infection, gender and height. Respir Med 2008; 102:962-969

12 Kim SH, Kim TH, Sohn JW, et al. Reference values and determinants of exhaled nitric oxide in healthy Korean adults. J Asthma 2010; 47:563-567

13 Ko FW, Leung TF, Wong GW, et al. Determinants of, and reference equation for, exhaled nitric oxide in the Chinese population. Eur Respir J 2013; 42:767-775

14 See KC, Christiani DC. Normal values and thresholds for the clinical interpretation of exhaled nitric oxide levels in the US general population: results from the National Health and Nutrition Examination Survey 2007-2010. Chest 2013; 143:107-116

15 Zhang YM, Lin JT. [Normal values and influencing factors of fractional exhaled nitric oxide for Chinese healthy adults]. Zhonghua Yi Xue Za Zhi 2012; 92:1971-1973

16 Brooks CR, Brogan SB, van Dalen CJ, et al. Measurement of exhaled nitric oxide in a general population sample: a comparison of the Medisoft HypAir FE(NO) and Aerocrine NIOX analyzers. J Asthma 2011; 48:324-328

17 Pellegrino R, Viegi G, Brusasco V, et al. Interpretative strategies for lung function tests. Eur Respir J 2005; 26:948-968

18 Rouatbi S, Alqodwa A, Ben Mdella S, et al. Fraction of exhaled nitric oxide (Fe(NO) ) norms in healthy North African children 5-16 years old. Pediatr Pulmonol 2012

19 Ben Saad H, Prefaut C, Tabka Z, et al. 6-minute walk distance in healthy North Africans older than 40 years: influence of parity. Respir Med 2009; 103:74-84

20 Brody DJ, Zhang X, Kit BK, et al. Reference values and factors associated with exhaled nitric oxide: U.S. youth and adults. Respir Med 2013; 107:1682-1691

21 American Thoracic S, European Respiratory S. ATS/ERS recommendations for standardized procedures for the online and offline measurement of exhaled lower respiratory nitric oxide and nasal nitric oxide, 2005. Am J Respir Crit Care Med 2005; 171:912-930

22 Kharitonov SA, Robbins RA, Yates D, et al. Acute and chronic effects of cigarette smoking on exhaled nitric oxide. Am J Respir Crit Care Med 1995; 152:609-612

23 Persson MG, Zetterstrom O, Agrenius V, et al. Single-breath nitric oxide measurements in asthmatic patients and smokers. Lancet 1994; 343:146-147

24 Jacinto T, Alving K, Correia R, et al. Setting reference values for exhaled nitric oxide: a systematic review. Clin Respir J 2012

25 Bodini A, Peroni D, Loiacono A, et al. Exhaled nitric oxide daily evaluation is effective in monitoring exposure to relevant allergens in asthmatic children. Chest 2007; 132:1520-1525

26 Verges S, Tonini J, Flore P, et al. Exhaled nitric oxide in single and repetitive prolonged exercise. J Sports Sci 2006; 24:1157-1163

27 Olin AC, Stenfors N, Toren K, et al. Nitric oxide (NO) in exhaled air after experimental ozone exposure in humans. Respir Med 2001; 95:491-495

28 Delfino RJ, Staimer N, Gillen D, et al. Personal and ambient air pollution is associated with increased exhaled nitric oxide in children with asthma. Environ Health Perspect 2006; 114:1736-1743

29 Leon de la Barra S, Smith AD, Cowan JO, et al. Predicted versus absolute values in the application of exhaled nitric oxide measurements. Respir Med 2011; 105:1629-1634

30 Vijverberg SJ, Koster ES, Koenderman L, et al. Exhaled NO is a poor marker of asthma control in children with a reported use of asthma medication: a pharmacy-based study. Pediatr Allergy Immunol 2012; 23:529-536

31 Perez-de-Llano LA, Carballada F, Castro Anon O, et al. Exhaled nitric oxide predicts control in patients with difficult-to-treat asthma. Eur Respir J 2010; 35:1221-1227

32 Smith AD, Cowan JO, Filsell S, et al. Diagnosing asthma: comparisons between exhaled nitric oxide measurements and conventional tests. Am J Respir Crit Care Med 2004; 169:473-478

33 Dupont LJ, Demedts MG, Verleden GM. Prospective evaluation of the validity of exhaled nitric oxide for the diagnosis of asthma. Chest 2003; 123:751-756

34 Komakula S, Khatri S, Mermis J, et al. Body mass index is associated with reduced exhaled nitric oxide and higher exhaled 8-isoprostanes in asthmatics. Respir Res 2007; 8:32

35 Barnes PJ, Dweik RA, Gelb AF, et al. Exhaled nitric oxide in pulmonary diseases: a comprehensive review. Chest 2010; 138:682-692

36 Petsky HL, Cates CJ, Li A, et al. Tailored interventions based on exhaled nitric oxide versus clinical symptoms for asthma in children and adults. Cochrane Database Syst Rev 2009:CD006340
