# Supplementary figures and images for: Fraction of Exhaled Nitric Oxide (FeNO) Norms in Healthy Tunisian Adults
Source: Biomed Res Int. 2014 Jun 3;2014:269670. doi: 10.1155/2014/269670 (PMC4065671; doi:10.1155/2014/269670)

## Slide 1
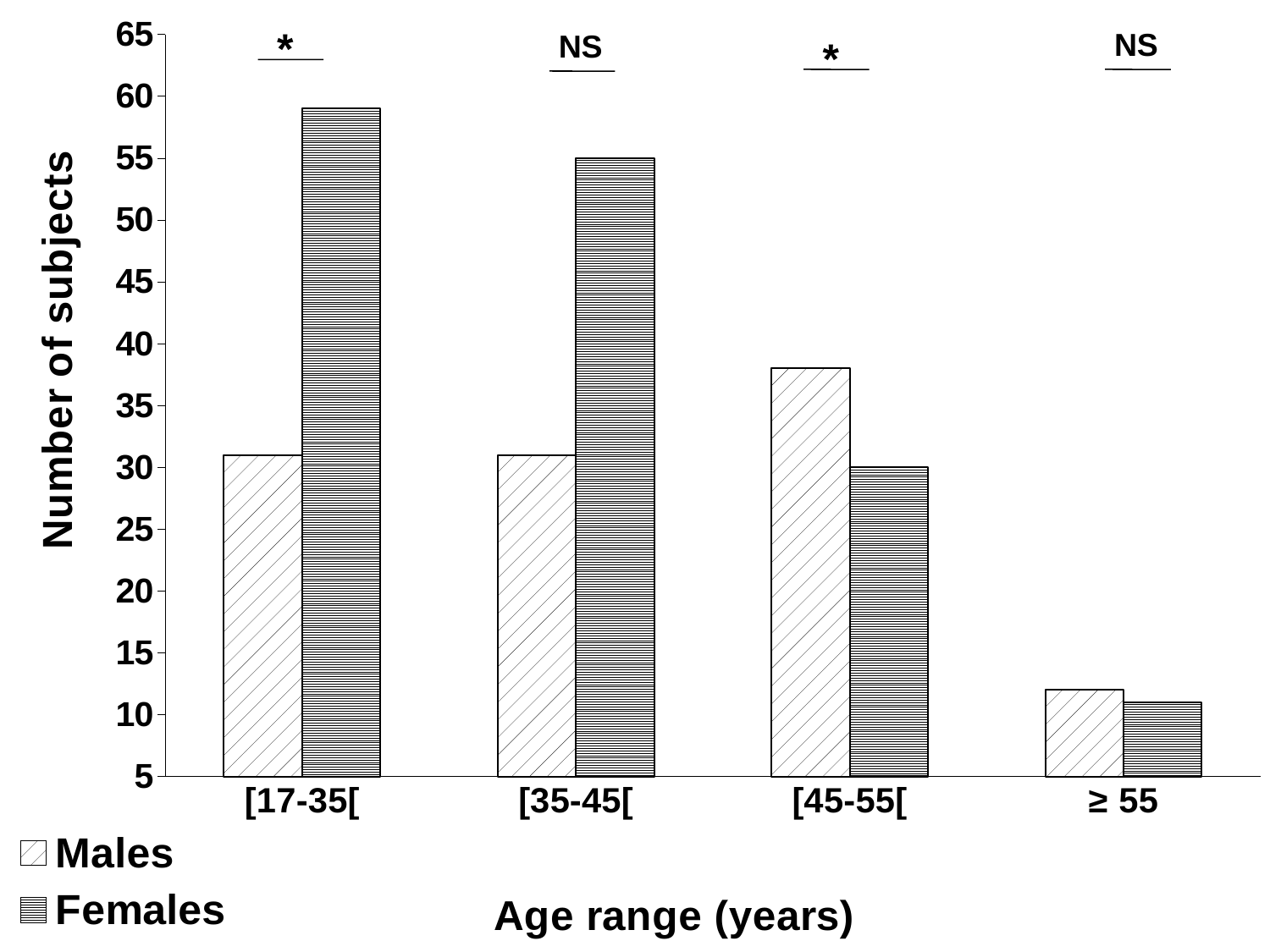

### Chart
| Category | Males | Females |
|---|---|---|
| [17-35[ | 31.0 | 59.0 |
| [35-45[ | 31.0 | 55.0 |
| [45-55[ | 38.0 | 30.0 |
| ≥ 55 | 12.0 | 11.0 |
*
NS
NS
*

Supplement: Supplementary file 2 [file 269670.f2.zip › 269670.f2/Figure_1_Supplementary_data_2738_931348.pptx]

## Slide 1
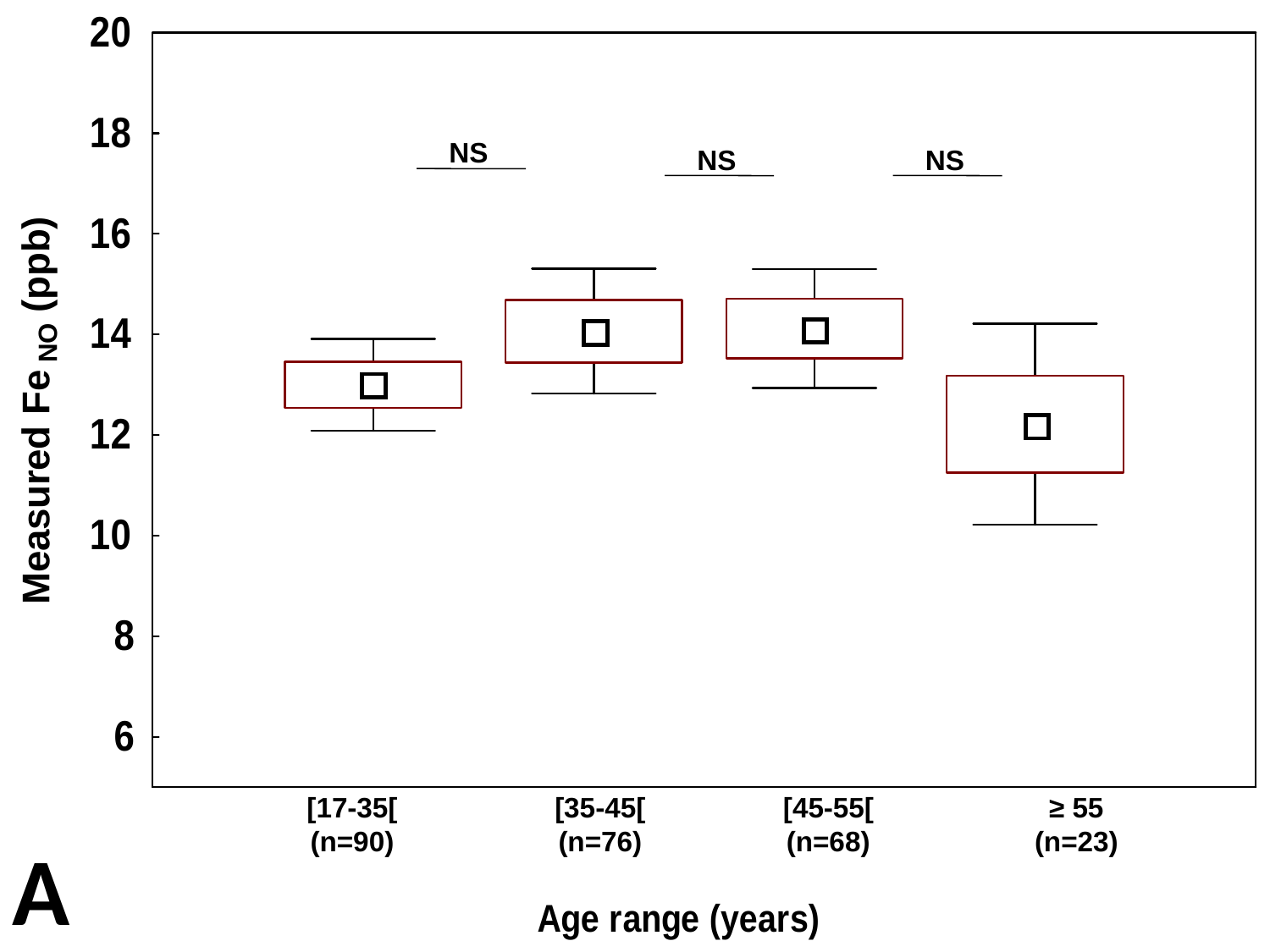

NS
NS
NS
[17-35[
(n=90)
[35-45[
(n=76)
[45-55[
(n=68)
≥ 55
(n=23)
A

## Slide 2
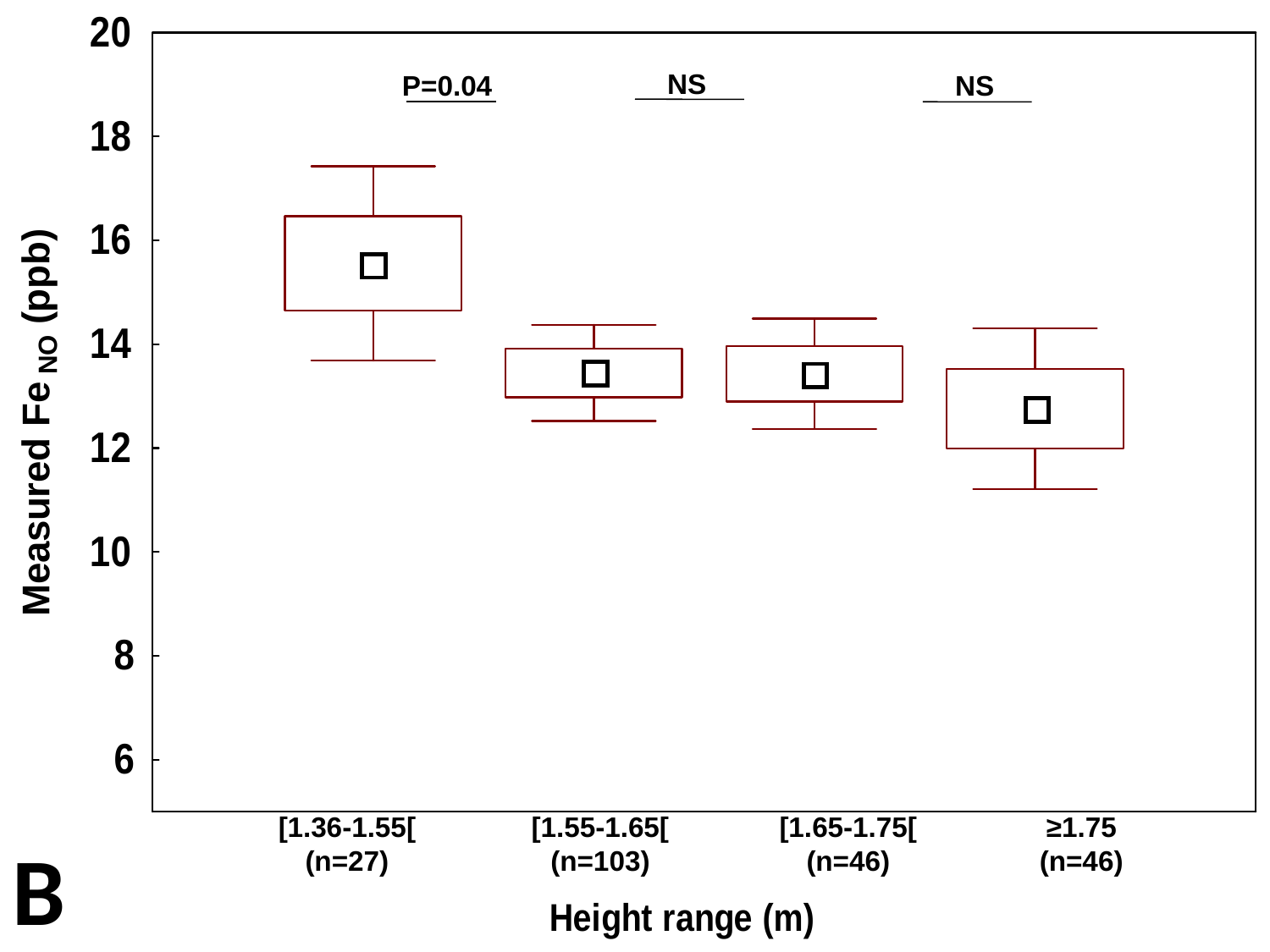

NS
P=0.04
NS
[1.36-1.55[
(n=27)
[1.55-1.65[
(n=103)
[1.65-1.75[
(n=46)
≥1.75
(n=46)
B

## Slide 3
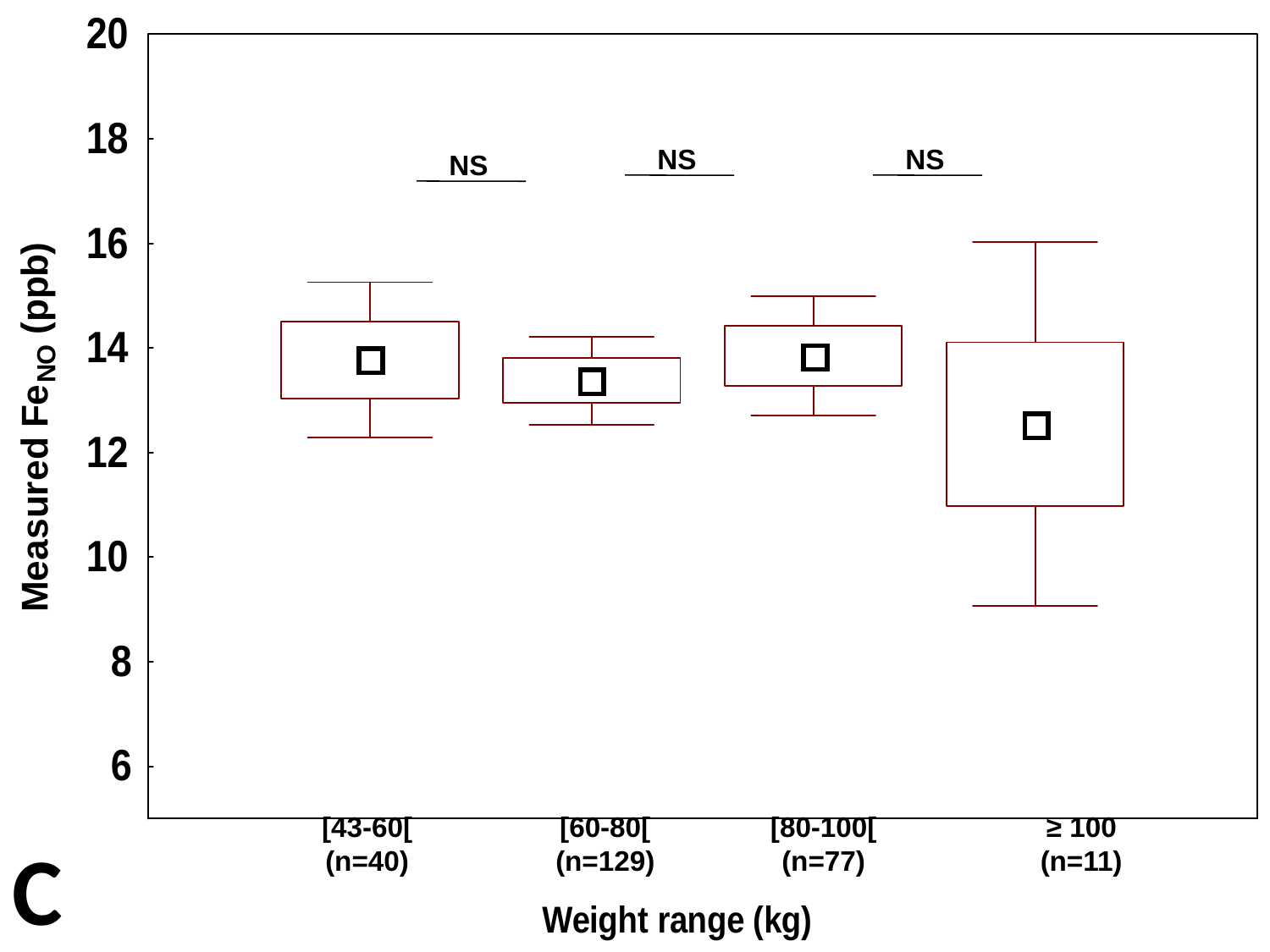

NS
NS
NS
[43-60[
(n=40)
[60-80[
(n=129)
[80-100[
(n=77)
≥ 100
(n=11)
C

Supplement: Supplementary file 2 [file 269670.f2.zip › 269670.f2/Figure_2_ABC_Supplementary_Data_2738_931346.pptx]

## Slide 1
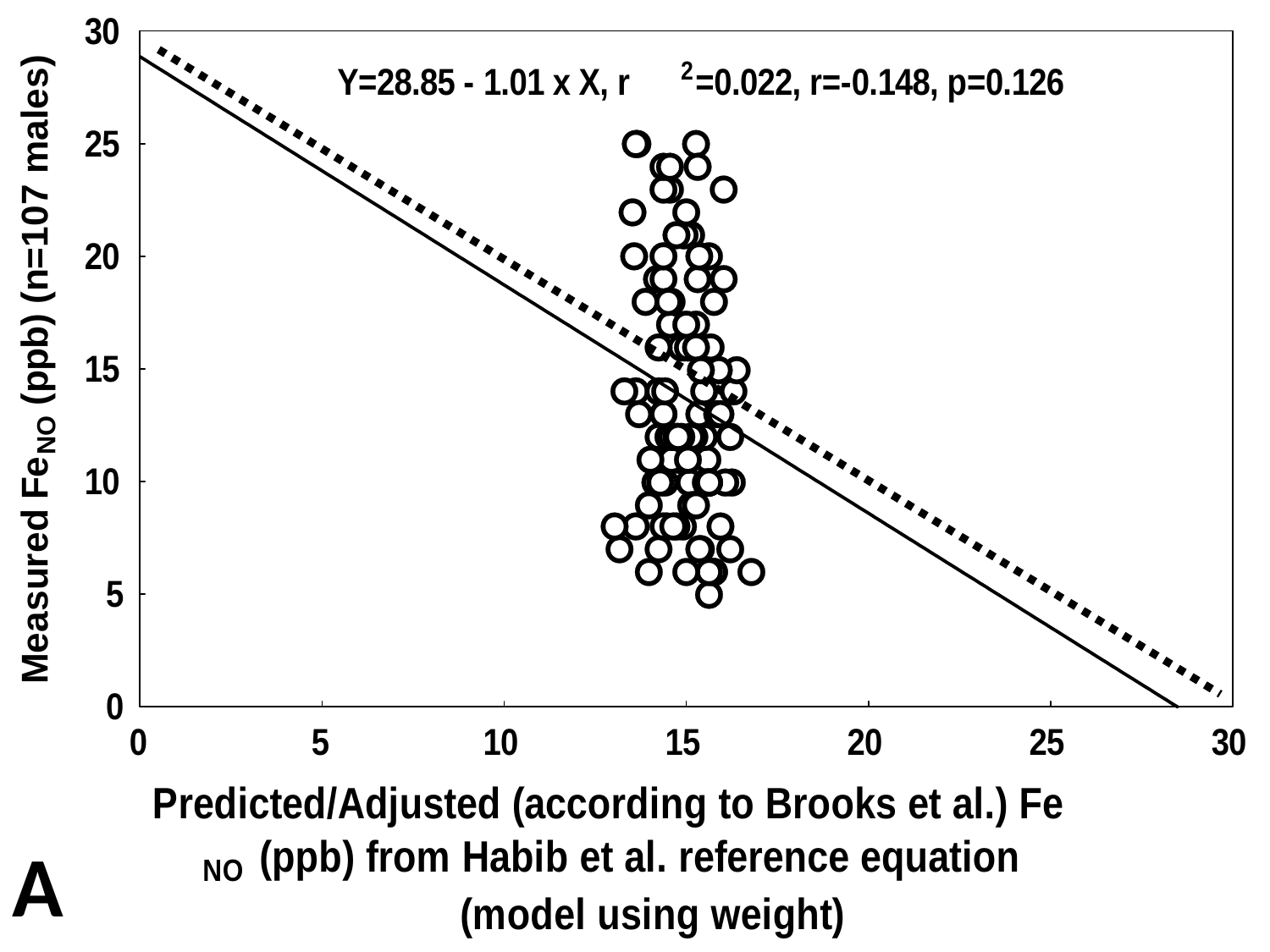

A

## Slide 2
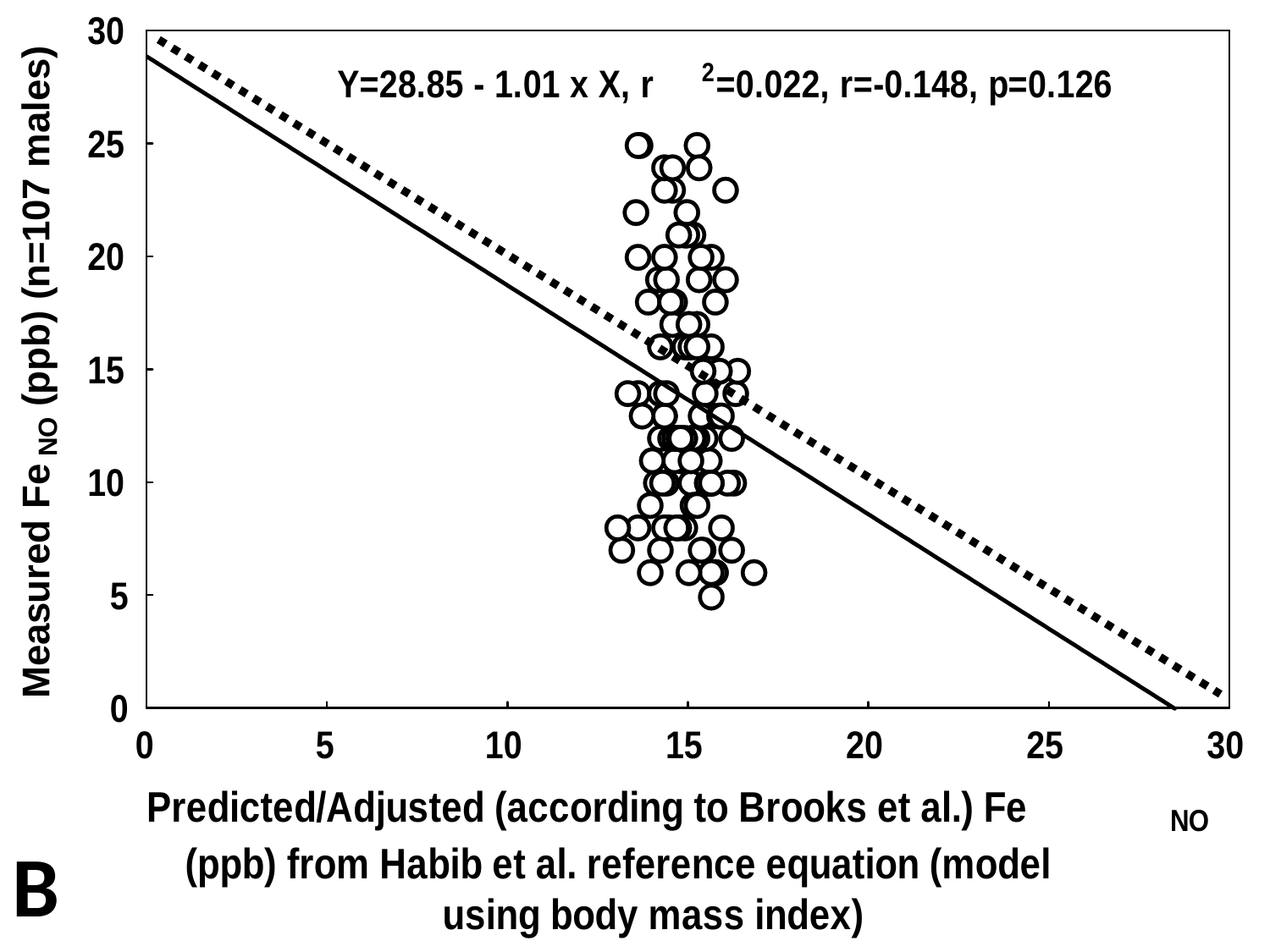

B

Supplement: Supplementary file 2 [file 269670.f2.zip › 269670.f2/Figure_3_ABC_supplementary_Data_2738_931345.pptx]

## Slide 1
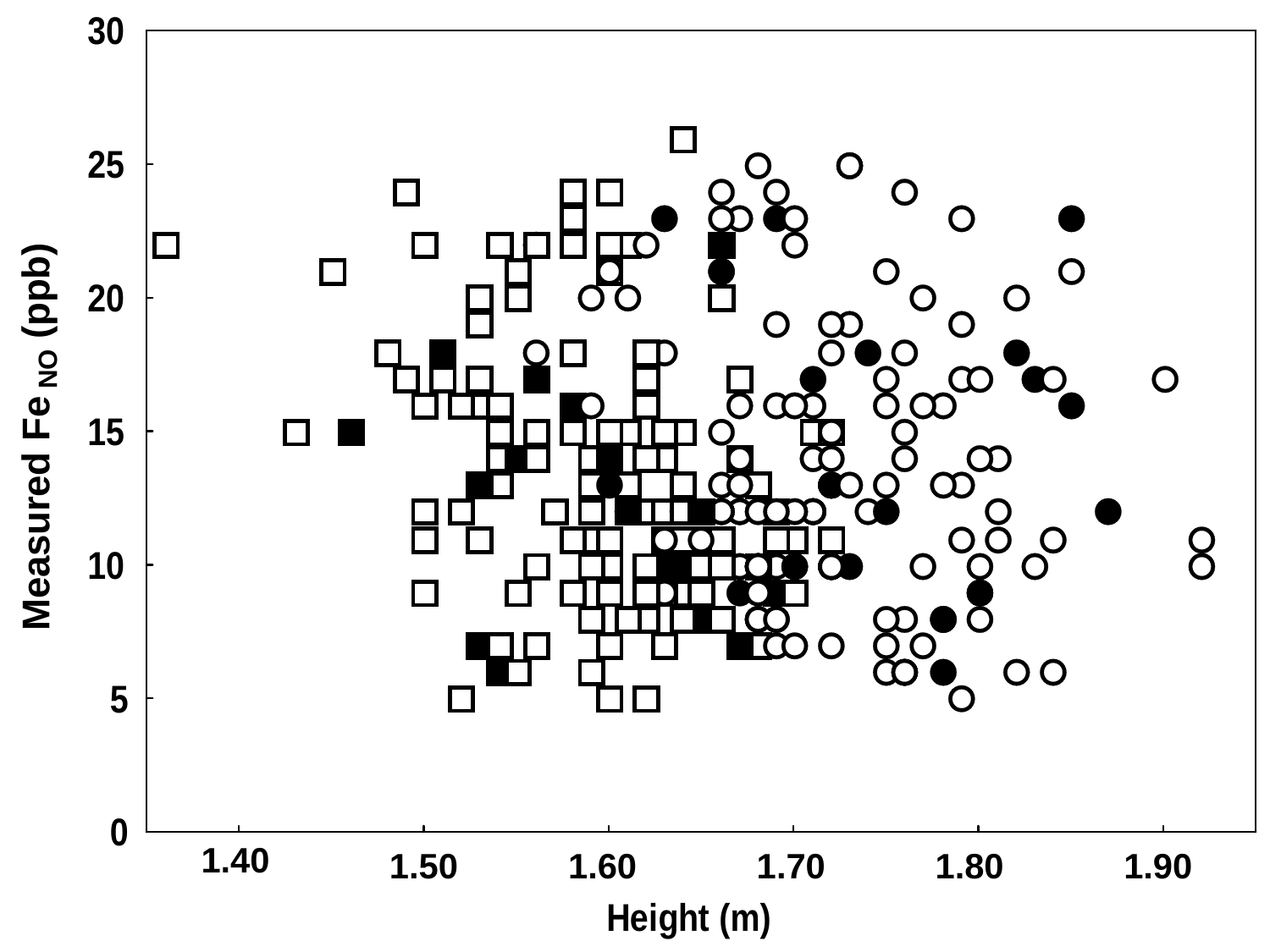

1.40
1.50
1.60
1.70
1.80
1.90

Supplement: Supplementary file 2 [file 269670.f2.zip › 269670.f2/Figure_4_supplementary_data_2738_931350.pptx]
